# From Descriptor Learning to Binding Stability: An Explainable Machine Learning Pipeline for EGFR Double-Mutant Inhibitor Discovery

**DOI:** 10.3390/ijms27167122

**Published:** 2026-08-08

**Authors:** Jurica Novak

**Affiliations:** Centre for Informatics and Computing, Ruđer Bošković Institute, Bijenička cesta 54, 10000 Zagreb, Croatia; jnovak@irb.hr

**Keywords:** EGFR, T790M/L858R mutant, drug design, molecular dynamics, machine learning, inhibitors, cancer development, drug resistance

## Abstract

Drug resistance arising during cancer development and progression remains a major challenge in the treatment of epidermal growth factor receptor (EGFR)-driven tumors, particularly those harboring the clinically relevant T790M/L858R double mutation. In this study, we developed an integrated computational workflow combining explainable machine learning, virtual screening, molecular dynamics simulations, and binding free-energy calculations to identify novel inhibitors of this drug-resistant EGFR variant. An XGBoost regression model was trained using scaffold-aware cross-validation, Bayesian hyperparameter optimization, and sequential feature selection, resulting in a compact model based on 16 molecular descriptors. The model demonstrated robust predictive performance on external validation data, while SHAP analysis identified descriptors related to the local electronic environment, fragment distribution, and molecular topology as the primary contributors to activity prediction. The optimized model was subsequently applied to screen compounds from the Enamine REAL database. Top-ranked candidates were evaluated using explicit-solvent molecular dynamics simulations and MM/GBSA binding free-energy calculations. Several compounds formed stable protein–ligand complexes and maintained key interactions with residues known to be important for EGFR inhibition, including Lys745, Met790, and Leu718. These results demonstrate that the proposed workflow can efficiently prioritize computational candidates of drug-resistant EGFR mutants and may support the development of new therapeutic strategies for overcoming resistance in EGFR-driven cancers.

## 1. Introduction

The epidermal growth factor receptor (EGFR; *ErbB1*; HER1), plays a central role among the ErbB family of receptor tyrosine kinases, which control the fate of the cell, including growth, survival, and differentiation [1,2,3,4]. Under physiological conditions, EGFR exists as an auto-inhibited monomer on the cell membrane. After the binding of a ligand, the receptor adopts an active conformation, facilitating the asymmetric interaction of the intracellular kinase domains, resulting in the trans-autophosphorylation of the *C*-terminal tail, leading to the activation of downstream pathways such as the Ras-MAPK and PI3K-Akt-mTOR pathways [3,4]. The overexpression of EGFR, usually due to amplification or somatic mutations, disrupts the balance of the signal, leading to the development of cancer, particularly non-small-cell lung cancer (NSCLC) [5].

The EGFR kinase domain consists of a bilobal structure, where the *N*-terminal and *C*-terminal lobes are connected by a short ‘hinge’ region [6,7,8]. The activation of the EGFR occurs through the movement of the αC-helix towards the intracellular end, resulting in the stabilization of the salt bridge between Lys745 and Glu762, leading to the binding of ATP–Mg^2+^ [9]. In the inactive ‘Src-like’ conformation, the αC-helix is displaced towards the extracellular end, resulting in the collapse of the activation loop [10].

Among the NSCLC population, the most common EGFR mutations are deletions in exon 19 and the L858R mutation in exon 21, leading to the constitutive activation of EGFR, resulting in the continuous activation of downstream pathways even without the binding of a ligand [11,12,13,14,15,16,17]. Although the disease initially responds to first-generation TKIs such as gefitinib and erlotinib, resistance develops inevitably. Among the 50–60% of cases, the T790M gatekeeper mutation accounts for the development of resistance through the increase in the affinity of the EGFR kinase for ATP, reducing the therapeutic window provided by the L858R mutation, which was previously thought to be steric hindrance by the T790 residue [18,19].

The emergence of secondary (T790M) and tertiary resistance (C797S) has brought the spotlight on the application of machine learning (ML) techniques for the discovery of fourth-generation drugs [8,20]. Recent studies reflect a trend toward the use of richer data with increased dimensions. For example, Nada et al. employed 9,000 compounds for the training of a random forest model for bioactivity prediction ($R_{val}^{2}=0.717$), which guided the synthesis of novel 4-anilinoquinazolines [21]. Zhang and Li developed a hybrid RF-RFE-SVM approach with exceptional performance for the prediction of fourth-generation drugs ($R_{test}^{2}=0.93$) [8]. The EGFR^*AP*^ tool, which is based on the extra trees regressor approach, showed a Pearson correlation coefficient of 0.82 on the test compounds [22].

From a chemometric perspective, the robustness of the developed models must be evaluated using the validation criteria suggested by Tropsha [23], Gramatica [24], and Roy [25]. It is interesting to note that while the developed models exhibit impressively high $R^{2}$ values, they do not provide a quantitative definition of the applicability domain (AD) of the model. Without the evaluation of the AD using tools such as Williams plots or leverage method, the statistical validity of the developed model is questionable.

Structure-based virtual screening (SBVS) combined with molecular dynamics (MD) is a key component of the modern computational toolkit for the discovery of EGFR inhibitors [26]. SBVS has focused on the discovery of allosteric site inhibitors with a binding site created by the movement of the *C*-helix [4]. For example, EAI045 is an allosteric inhibitor that targets the EGFR mutant. MD is a vital tool for the simulation of the behavior of mutant EGFRs. For example, Park et al. showed the effect of the T790M mutation on the binding affinity of the inhibitor using MD simulations. The T790M mutant shows a 2–4 kcal/mol penalty on inhibitor binding due to the movement of the αC-helix [27]. Fast MD simulations (up to 500 ns) were employed to elucidate the allosteric activation mechanism of rare mutant EGFRs, such as the D761N mutant, which creates a hydrogen-bond network between the αC-helix and the activation loop [10].

Recent advances in computational drug discovery have increasingly incorporated machine learning, network-based representations, and generative models to explore chemical and biological space beyond conventional virtual screening pipelines. In particular, network embedding approaches have been applied to drug–disease association prediction by learning low-dimensional representations of heterogeneous biomedical graphs [28], while deep generative frameworks enable de novo molecular design with optimized physicochemical properties [29]. These developments complement traditional structure-based drug discovery workflows by expanding the methodological landscape for identifying and prioritizing bioactive compounds.

In parallel, classical machine learning approaches, including support vector machines, random forests, and neural networks, have been widely applied to predict inhibitory activity against clinically relevant EGFR mutants such as the L858R/T790M double mutation using molecular descriptors and fingerprints [26,30]. Agarwal et al. employed a general anticancer machine learning model to prioritize potential phytochemicals, which were subsequently refined through molecular docking and molecular dynamics simulations to identify promising lead compounds [26,31]. Similarly, Saini et al. developed a classification model using a 100 nM activity threshold to distinguish active from inactive EGFR inhibitors, demonstrating the utility of strict potency cutoffs in binary activity prediction [30]. Furthermore, Chang et al. constructed a predictive model specifically for the EGFR L858R/T790M/C797S triple mutant, trained exclusively on a curated dataset of 221 known inhibitors targeting this clinically resistant variant [20]. In addition, Sun et al. proposed a multitask learning framework capable of simultaneously modeling inhibitory activity across multiple EGFR mutant forms, thereby improving generalization across related kinase targets [32].

In contrast, the present study focuses on an integrated structure-based virtual screening pipeline combining ligand-based filtering, molecular docking, and molecular dynamics simulations to identify and prioritize novel EGFR T790M/L858R inhibitors from large chemical libraries.

## 2. Results and Discussion

### 2.1. Dataset Curation, Scaffold-Aware Splitting, and Feature Selection

The initial EGFR activity dataset obtained from ChEMBL contained 18,105 entries. Sequential curation steps—including the standardization of units, the selection of compounds active against the L858R/T790M double mutant, the removal of ambiguous or non-nanomolar activity measurements, the resolution of duplicate entries, salt stripping, and the exclusion of out-of-range annotations—yielded a high-quality collection of 1148 unique molecules. A histogram of the pIC_50_ distribution for the complete curated dataset is provided in the Supporting Information (Figure S2), illustrating the range and frequency of activity values used for subsequent descriptor calculation and model training.

Scaffold analysis of the curated set identified 489 distinct Murcko scaffolds. Of these, 827 molecules belonged to 168 scaffolds with two or more representatives, while the remaining 321 molecules corresponded to unique scaffolds. To generate independent training and validation sets, 917 molecules were assigned to the training set and 231 molecules to the independent validation set. This split preserved the proportion of scaffolds with multiple representatives between the training and validation sets, maintaining chemical diversity comparable to the original dataset. Single-molecule scaffolds were randomly allocated to either set.

The same scaffold-aware approach was applied when partitioning the training set into folds for five-fold cross-validation. In this case, scaffolds containing five or more molecules were monitored to ensure balanced representation across folds, preventing the over-representation of dominant scaffolds in any single fold.

Molecular descriptors were computed using the combined RDKit and Mordred workflows, capturing structural and physicochemical properties in one-, two-, and three-dimensional representations. After the removal of descriptors containing missing or non-numerical values, the dataset comprised 1649 fully numeric features. To reduce dimensionality and improve model interpretability, a multi-stage feature selection protocol was applied. Initial filtering steps removed sparse and low-variance descriptors, followed by elimination of highly correlated features based on Spearman correlation analysis. This reduced the descriptor set to 393 chemically meaningful features. A subsequent application of the RReliefF algorithm prioritized descriptors based on their predictive relevance for pIC_50_ values, resulting in a subset of 50 top-ranking features. The RReliefF procedure was carried out using an extreme gradient boosting (XGBoost) regression model configured with the optimized hyperparameter set obtained through Optuna-based optimization, ensuring that feature relevance was assessed in the context of the final predictive model. Feature importance scores were first calculated independently within each cross-validation fold and then averaged across folds to ensure robust ranking. Based on these aggregated scores, the top 50 descriptors were selected for further analysis and model development. A heatmap illustrating the relative importance of the top 25 descriptors across all folds is provided in the Supporting Information (Figure S3), where the color intensity represents the RReliefF score in each fold.

Finally, sequential feature selection (SFS) was performed in combination with XGBoost hyperparameter optimization to systematically evaluate the dependence of model performance on the number of retained descriptors. The highest mean cross-validation score was achieved with a subset of 31 descriptors, yielding an average score of –0.882. A more compact model constructed using 16 descriptors produced a closely comparable mean score of –0.892, corresponding to a marginal difference of 0.010 (approximately 1.1% relative deviation).

Considering the negligible performance decrease and the nearly twofold reduction in input dimensionality, the 16-descriptor model was selected for all subsequent analyses. This more parsimonious representation decreases model complexity, lowers the risk of overfitting, and improves interpretability without materially compromising predictive accuracy. From a practical standpoint, descriptor reduction also enhances computational efficiency and robustness when applying the model to ultra-large virtual screening libraries, where scalability and stable generalization to previously unseen chemical space are critical. The dependence of model score on the number of selected features is illustrated in the Supporting Information (Figure S4), which presents the feature count versus cross-validation score obtained during the SFS procedure.

### 2.2. Machine Learning Model Performance

For the final model development stage, three regression algorithms were considered: extreme gradient boosting (XGBoost), support vector machines (SVMs), and random forest (RF). Each algorithm was trained using the selected feature subset comprising 16 descriptors. To ensure a fair and directly comparable evaluation, the same scaffold-aware five-fold cross-validation splits were employed across all models and feature configurations.

Hyperparameter optimization for XGBoost, SVM, and RF was performed independently using the Optuna framework. For each Optuna trial, models were trained and evaluated across all cross-validation folds, and performance metrics were collected separately for the training, testing and validation partitions. The optimization objective was designed to jointly account for predictive accuracy and generalization behavior by penalizing overfitting.

Specifically, the mean root-mean-square error (RMSE) was computed across folds for both training and test sets. An overfitting gap was defined as the difference between test and training RMSE values. The final scalar objective optimized by Optuna was defined as

(1) $$\mathrm{Score}=-\left({\mathrm{RMSE}}_{\mathrm{test}}+{\left({\mathrm{RMSE}}_{\mathrm{test}}-{\mathrm{RMSE}}_{\mathrm{train}}\right)}^{2}\right),$$

where ${\mathrm{RMSE}}_{\mathrm{train}}$ and ${\mathrm{RMSE}}_{\mathrm{test}}$ denote the mean training and test RMSE values averaged across cross-validation folds. This formulation favors models with low prediction error while explicitly penalizing large discrepancies between training and testing performance, thereby promoting robust generalization.

For each algorithm and feature set, the hyperparameter combination yielding the highest optimization score was selected. Final models were subsequently retrained using these optimized hyperparameters on the full training dataset, and their predictive performance was assessed on the independent validation set. The corresponding hyperparameter search spaces and the optimized values identified for each model are summarized in Table S1 in the Supporting Information.

The Optuna-driven hyperparameter optimization revealed a structured and non-random performance landscape across the explored search space, indicating effective convergence of the Bayesian optimization procedure. Model performance was not uniformly distributed but instead clustered within a well-defined region of the parameter domain, suggesting sensitivity to specific regularization and tree-complexity settings rather than stochastic variability. The absence of multiple equivalent optima further supports the stability of the optimization process and the robustness of the selected configuration.

**Figure 1:**
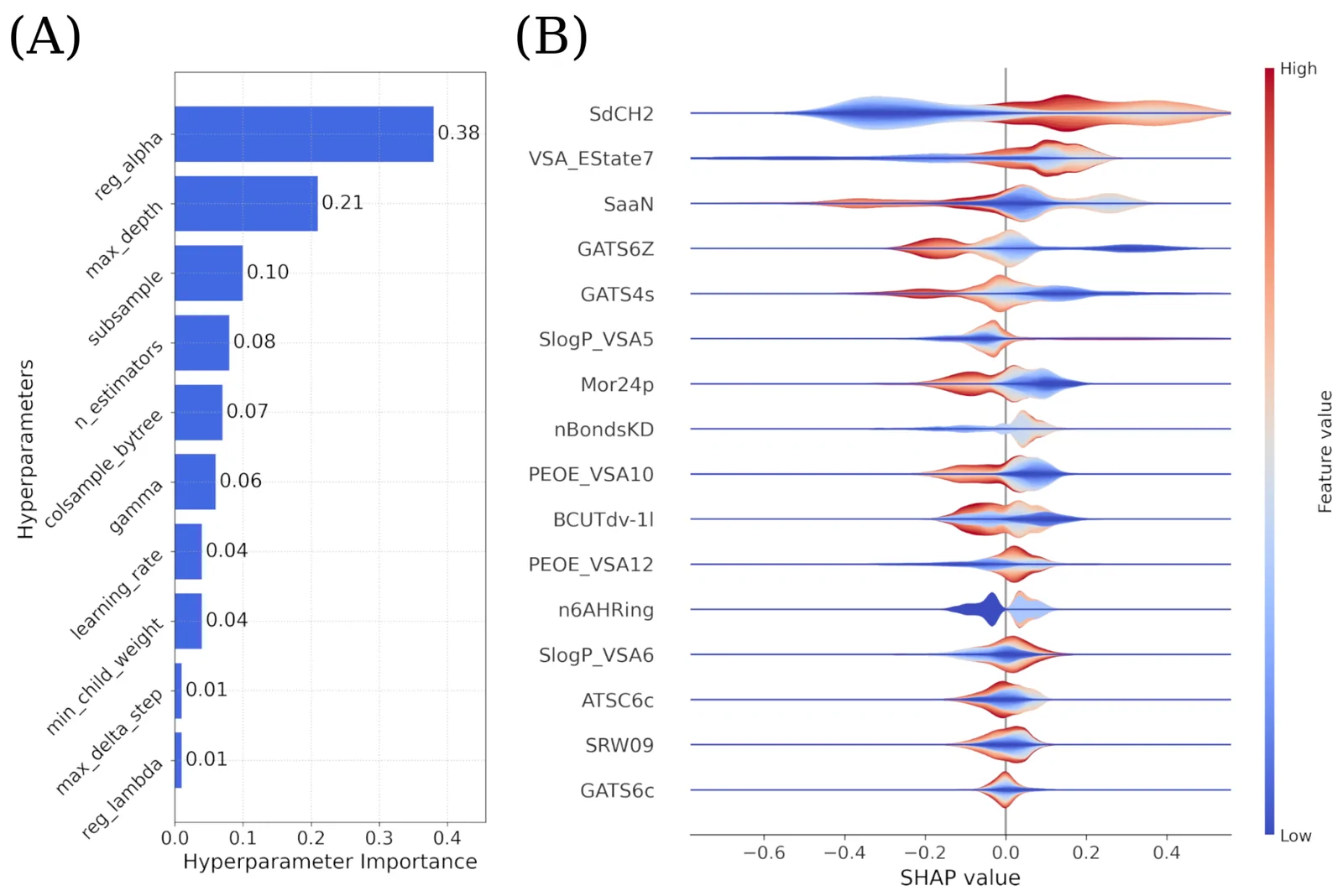
Global feature importance ranking derived from the trained XGBoost model (**A**). SHAP summary (violin) plot illustrating the distribution and magnitude of feature contributions to model predictions, with color indicating feature value intensity (**B**).

Analysis of the importance of the hyperparameters, based on the mean absolute SHAP values, indicates that reg_alpha and max_depth have the most impact on the XGBoost model predictions. The mean (|SHAP|) for reg_alpha and max_depth was approximately 0.38 and 0.21 pIC_50_ units, respectively. This further emphasizes the importance of L1 regularization and tree depth in preventing overfitting, which allows the model to learn non-linear structure–activity relationships. The subsample parameter also has a moderate impact on the model predictions, with a mean (|SHAP|) of approximately 0.10 pIC_50_ units. The impact of the other hyperparameters, such as n_estimators, colsample_bytree, and gamma, was somewhat lower, while the impact of min_child_weight, learning_rate, reg_lambda, and max_delta_step was minimal, with a mean (|SHAP|) < 0.05 pIC_50_ units. The importance of the hyperparameters is depicted in Figure 1A. The results suggest that the model’s ability to accurately predict EGFR inhibitory activity is mainly driven by the balance between the regularization strength and tree depth, with the other hyperparameters playing a role in fine-tuning the model’s stability.

The predictive performance of the three regression models—XGBoost, RF, and SVM—was systematically evaluated across cross-validation folds, the full training set, and an independent validation set (Table 1). During scaffold-aware five-fold cross-validation (Figure S5), XGBoost was found to perform better than the other two algorithms, RF and SVM, in all the performance metrics. XGBoost was found to have the least average prediction error on the test folds, with an RMSE of 0.868±0.035, whereas the average prediction error was found to be greater for RF and SVM, with an RMSE of 0.904±0.032 and 0.911±0.056, respectively. XGBoost was also found to have the highest explained variance and linear correlation with the predicted and experimental values of pIC_50_. The performance gap between the training and the test folds was found to be smaller for XGBoost, which indicated that the model had not overfit the data, despite its flexibility and nonlinearity. This was due to the use of the Optuna-based hyperparameter optimization strategy, where the generalization gap was penalized. RF and SVM were found to have lower training performance and a comparable train-test gap with a high average prediction error, which indicated that the models had a lower ability to generalize.

Evaluation on the full training set further confirmed the robustness of the XGBoost model, which achieved a low prediction error (RMSE = 0.617) and strong agreement with experimental pIC_50_ values, indicating effective learning of the underlying structure–activity relationships. While RF and SVM also captured general trends, their higher errors suggest a reduced ability to fully exploit the selected descriptor space.

**Figure 2:**
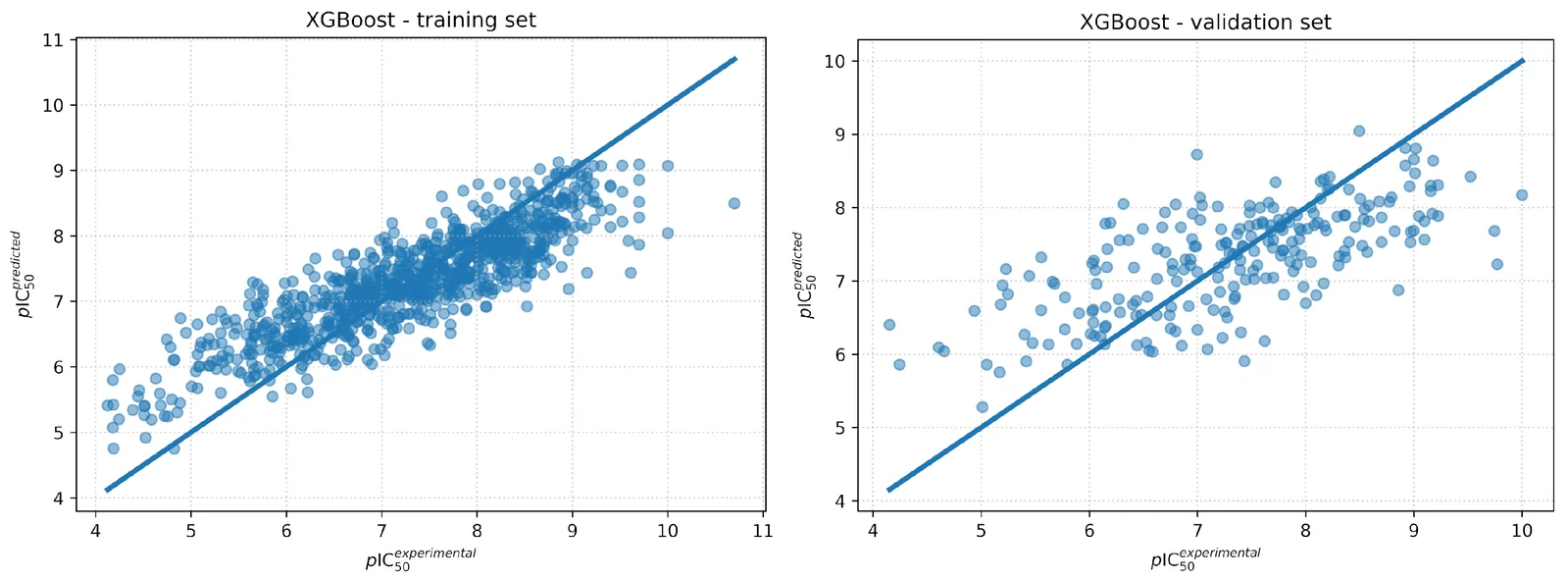
Comparison of experimentally measured versus XGBoost-predicted pIC_50_ values. (**Left panel**) predictions on the cross-validation test folds. (**Right panel**) predictions on the independent external validation set. The diagonal line represents perfect agreement between experimental and predicted values, illustrating model performance and generalization to unseen chemical space.

The independent validation set is crucial for virtual screening, as it evaluates model performance on unseen chemical structures. On this dataset, XGBoost outperformed the other algorithms, achieving the lowest error (RMSE = 0.830) and the highest linear correlation (PCC = 0.682), thereby demonstrating robust extrapolative capacity (Figure 2). In comparison, RF and SVM exhibited lower correlation and higher errors.

To ensure a realistic assessment of robustness, we employed rigorous scaffold-aware cross-validation alongside this external validation. The expected increase in RMSE from 0.617 on the training set to 0.830 on the independent set represents a controlled decrease in predictive performance when transitioning to novel chemotypes. The absence of a drastic drop in accuracy confirms that the model does not suffer from overfitting, validating our regularization strategy and parameter optimization. Finally, to ensure the reliable translation of these findings, we explicitly defined the model’s applicability domain, restricting future predictions to the chemical space adequately represented in our training data and preventing unreliable predictions on structurally dissimilar molecules.

The SHAP feature importance analysis suggests that the XGBoost model utilizes a combination of fragment-based, surface–electronic, and topological autocorrelation properties to describe the molecular basis of EGFR inhibitory activity (Figure 1B). Only a small number of these descriptors have a significant and consistent impact on the predicted activity, with SdCH_2_, VSA_EState7, and SaaN identified as the most important.

Among these, the fragment-count descriptor SdCH_2_, which quantifies the dispersion of methylene (–CH_2_–) groups and reflects the distribution of aliphatic carbon fragments, has the highest SHAP values. High values of both SdCH_2_ and the surface-area descriptor VSA_EState7 (representing the van der Waals surface area within a defined electrotopological interval) are associated with an increase in predicted pIC_50_ values. Conversely, an increase in SaaN values, which captures the presence of aromatic nitrogen atoms, results in a decrease in predicted activity. This highlights the substantial influence of molecular size, aliphatic distribution, and heteroatom placement on target engagement within the EGFR ATP-binding pocket.

Similarly, surface-area descriptors such as SlogP_VSA5 and SlogP_VSA6 describe the critical balance between hydrophobic and polar molecular surface regions. Along with topological autocorrelation descriptors—including GATS4s and GATS6Z (computed at topological lags of four and six using intrinsic state and atomic number as weighting schemes)—these features show more balanced SHAP value distributions around zero. This indicates subtler, conformation-dependent and lipophilicity-driven effects that fine-tune, rather than dominate, model predictions by capturing the spatial distribution of atomic properties. Secondary contributors, such as Mor24p, nBondsKD, and the PEOE_VSA family, exert moderate but non-negligible impact, reflecting the influence of overall molecular shape and polar surface.

Although these selected descriptors characterize intrinsic ligand properties rather than the EGFR T790M/L858R mutant itself, they encode physicochemical properties highly relevant for kinase inhibitor recognition. Their mechanistic relevance is further supported by subsequent molecular dynamics simulations and interaction fingerprint analyses, which reveal persistent interactions with key residues within the ATP-binding pocket.

At present, no broadly validated predictive models specifically addressing dual-mutant EGFR inhibition are available for direct benchmarking. A recent study described the development of a machine learning model trained on a relatively small dataset of 38 compounds to predict allosteric inhibition of the inactive state of EGFR T790M/L858R mutants [33]. Among the five evaluated algorithms, XGBoost achieved the lowest RMSE on the training set (0.0004), while the corresponding test set RMSE was reported as 0.537. The pronounced discrepancy between training and test errors suggests limited generalization capacity, which may be attributable to the restricted dataset size and the inherent challenges of modeling such a narrowly defined chemical space. In addition, an explicit applicability domain analysis was not reported, making it difficult to assess the reliability of extrapolations beyond the training compounds.

### 2.3. Virtual Screening of Enamine Database

**Figure 3:**
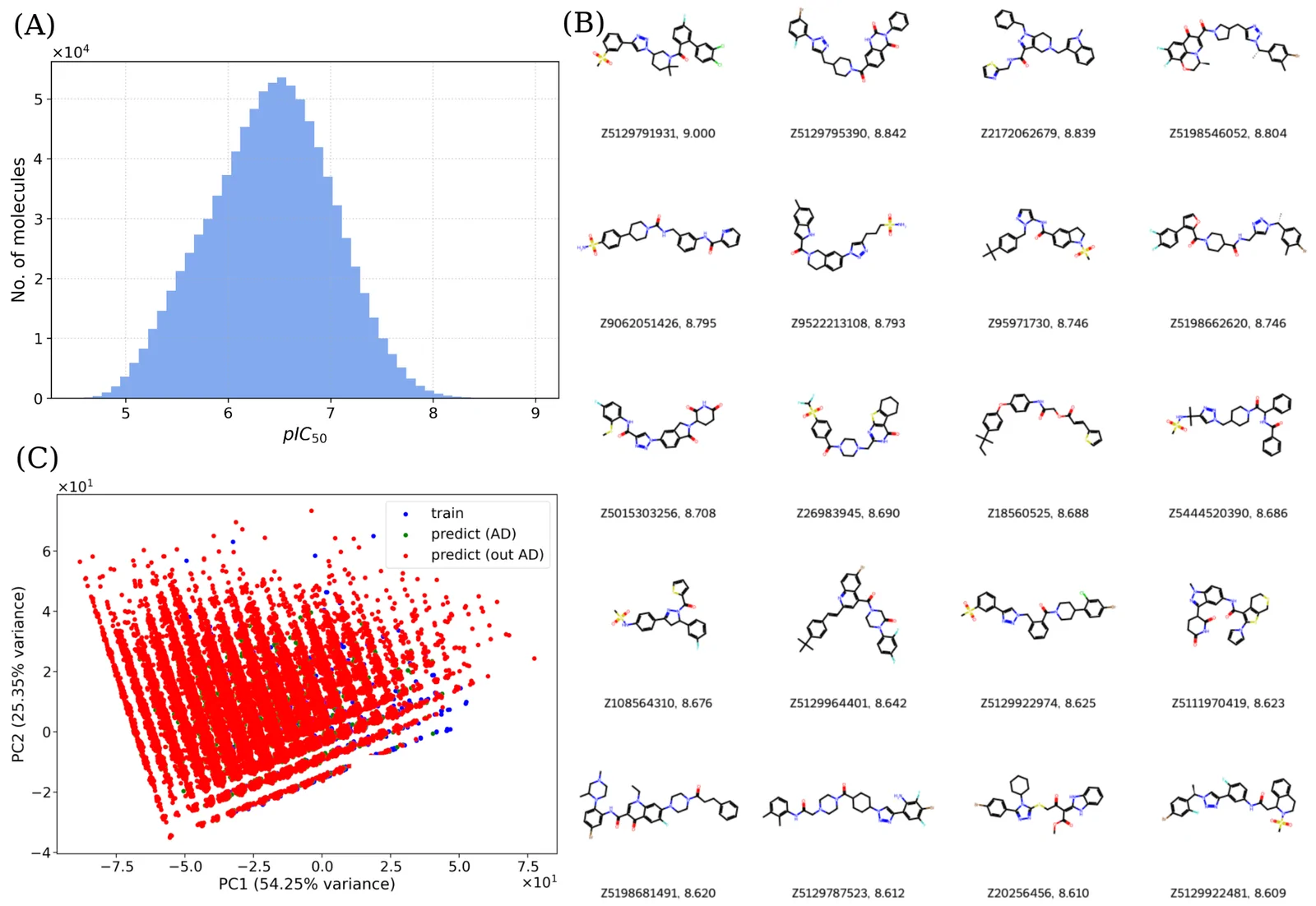
Virtual screening results for Enamine REAL database compounds within the defined applicability domain. (**A**) Distribution of predicted pIC_50_ values for all compounds retained after applicability domain filtering. (**B**) Two-dimensional structures, compound identifiers, and predicted pIC_50_ values for the 20 top-ranked candidates with the highest predicted activity. (**C**) Two-dimensional PCA projection of representative screened compounds (Z26476809–Z27248882) relative to the training chemical space. Compounds classified as inside or outside the applicability domain were identified using the Mahalanobis distance in the original descriptor space; the PCA representation is provided for visualization purposes only.

Virtual screening was conducted on a large subset of the Enamine REAL compound collection. The distribution of predicted activities is summarized in Figure 3A. Across the entire screened set, the mean predicted pIC_50_ was 6.41, and a median of 6.43. Predicted values ranged from 4.50 to 9.00, with 1976 compounds exceeding a predicted pIC_50_ of 8.0. For context, the independent validation set exhibited experimental pIC_50_ values spanning 4.2 to 10.0, whereas the XGBoost model predicted a slightly narrower range of 5.3 to 9.0. This contraction at the extremes is consistent with the limited number of compounds exhibiting very low or very high activity in the training data. A detailed examination across five cross-validation folds confirms this trend: the number of extreme predicted values (below the 5th percentile, <5.259, or above the 95th percentile, >9.000) remained low, ranging from 15 to 21 compounds per fold.

Inspection of the top-ranked compounds (predicted activity range ∼8.60–9.00) reveals a pronounced structural convergence toward elongated, modular architectures composed of two to three aromatic or heteroaromatic rings connected through short, heteroatom-rich linkers (Figure 3B). The majority of high-scoring molecules contain biaryl or aryl–heteroaryl frameworks incorporating *sp*^2^ nitrogen atoms, frequently combined with amide functionalities. This recurrent motif suggests that the predictive model favors chemotypes capable of simultaneously engaging in π–π interactions and directional hydrogen bonding. The consistent presence of carbonyl-containing linkers further indicates that hydrogen-bond acceptor geometry and partial conformational preorganization contribute significantly to the predicted activity landscape.

A notable enrichment of sulfonamide and sulfonyl-substituted fragments is observed among the highest-ranked candidates. These groups introduce strong hydrogen-bond acceptor sites and increase local polarity while preserving overall scaffold rigidity. In addition, halogen substitution–predominantly chlorine and bromine–appears frequently on peripheral aromatic rings. Such substitutions likely modulate electronic density and lipophilicity, potentially enhancing hydrophobic pocket occupancy and enabling favorable halogen-bond or dispersion-driven interactions. The combined presence of aromatic anchoring elements and polar linkers suggests a binding environment that accommodates both hydrophobic stacking interactions and specific electrostatic contacts.

In order to assess the structural redundancy of the highest-ranked candidates, pairwise Tanimoto similarities were computed using Morgan fingerprints (radius = 2, 2048 bits, Figure S6). The resulting similarity matrix reveals predominantly low off-diagonal values, with most pairwise coefficients residing in the low similarity regime. Quantitatively, only 17 pairwise comparisons out of the top 50 molecules exhibit a Tanimoto similarity above 0.4, while all remaining pairs fall below this threshold, indicating generally low structural redundancy within the selected set.

Apart from a limited number of localized clusters exhibiting moderate similarity, the majority of compounds display weak mutual resemblance, indicating that the top-ranked set does not collapse into a single scaffold family. Instead, the heat map suggests a structurally diverse ensemble of chemotypes occupying distinct regions of fingerprint space, supporting the conclusion that the model-based prioritization captures multiple independent chemical series rather than a single dominant scaffold cluster.

From the full library (>4,000,000 compounds), activity predictions were successfully generated for a total of 901,654 compounds. The reduced number of screened molecules relative to the nominal size of the library resulted from two deliberate and conservative filtering steps applied prior to prediction. First, only compounds with fully defined numerical values for all 16 selected molecular descriptors were retained. Molecules for which at least one of the required descriptors was undefined or non-numeric were excluded from further analysis. This conservative criterion was adopted to avoid implicit imputation or extrapolation within descriptor space and to ensure strict compatibility with the trained XGBoost model.

Second, an explicit AD filter was applied before property prediction. Each candidate molecule was evaluated by calculating its Mahalanobis distance in the original 16-dimensional descriptor space relative to the training set. Compounds with Mahalanobis distances below the 95% confidence threshold were considered to lie within the model’s applicability domain and were retained for further analysis, whereas compounds exceeding this threshold were excluded from subsequent virtual screening. For visualization purposes, the resulting AD classification was projected onto a two-dimensional PCA representation of the chemical space (Figure 3C). This step ensured that predictions were restricted to regions of chemical space for which the model is expected to be reliable, thereby minimizing the risk of uncontrolled extrapolation.

### 2.4. Molecular Dynamics Simulations

The 50 PAINS-free compounds with the highest machine learning scores were advanced to the structure-based refinement stage involving explicit-solvent molecular dynamics simulations. Each protein–ligand complex was subjected to a standard preparation protocol consisting of energy minimization, gradual heating, and density equilibration prior to production dynamics. During the 50 ns production phase, the geometry of the solvated complex was recorded at regular intervals under periodic boundary conditions to ensure consistent sampling of conformational space.

Binding free energies were estimated for 250 uniformly distributed snapshots extracted along each trajectory using the MM/GBSA approach implemented in Amber, employing the GBneck2 implicit solvent model [34]. Entropic contributions were not included; therefore, the reported values reflect the enthalpic component of binding within the MM/GBSA framework. Based on the resulting $\Delta G_{\mathrm{bind}}$ estimates, the complexes were ranked, and the nine ligands exhibiting the most favorable binding energies were selected for extended simulations (Figure S7). This selection was performed as a prioritization step within the MM/GBSA-derived ranking scheme rather than as an absolute thermodynamic criterion, given the known approximations associated with implicit-solvent free energy estimations. To reduce noise arising from end-point free energy fluctuations and to enhance the robustness of candidate selection, ranking was based on averages computed over uniformly sampled trajectory snapshots.

For these top-ranked systems, three independent 300 ns production trajectories were performed using distinct initial Maxwell–Boltzmann velocity assignments to enhance statistical robustness. The replicate trajectories were concatenated for analysis, and structural stability was assessed through root-mean-square deviation (RMSD), radius of gyration ($R_{g}$), and root-mean-square fluctuation (RMSF) calculations. To facilitate a consistent and statistically robust analysis, the three independent 300 ns molecular dynamics replicates generated for each system were concatenated into a single continuous trajectory prior to post-processing. MM/GBSA binding free energy profiles were additionally evaluated over the extended trajectories to examine energetic stability and convergence behavior. An identical simulation and analysis protocol was applied to the apo EGFR T790M/L858R mutant, as well as to the mutant complexed with osimertinib (AZD9291), an oral, irreversible, and mutant-selective EGFR inhibitor, which served as a reference system.

Among the investigated compounds, three ligands—Z5129795390, Z5129787523, and Z5129914621—were identified as the top computationally identified hit candidates following rigorous statistical evaluation of MM/GBSA binding free energies obtained from independent 300 ns triplicate simulations. These candidates exhibit consistently robust non-covalent target engagement within the binding site, yielding bootstrap mean binding free energies that are highly comparable to or slightly lower than the initial, pre-reactive binding pose of the reference inhibitor AZD9291 (Table S2). In particular, Z5129795390 demonstrated the strongest overall performance, combining a highly favorable binding free energy (BootMean=−49.21 kcal mol^−1^) with low inter-replica variability (2.8 kcal mol^−1^) and adequate effective sampling ($N_{\mathrm{eff}}=19$). Similarly, Z5129787523 showed good statistical robustness, characterized by the high effective sample size ($N_{\mathrm{eff}}=24$) and 3.1 kcal mol^−1^ confidence interval, suggesting well-converged sampling and stable ligand–protein interactions throughout the simulations. Although Z5129914621 displayed slightly higher uncertainty, its consistently favorable binding free energy and acceptable replica agreement support its classification as a reliable high-affinity binder.

Importantly, ligand prioritization was not based solely on mean MM/GBSA energies but additionally incorporated multiple statistical quality criteria, including sampling convergence, temporal correlation, and reproducibility across independent simulations (Figure S8). In particular, ligands exhibiting strongly favorable mean binding energies (e.g., Z5198681491) were excluded from final selection due to elevated inter-replica variability and reduced effective sample sizes, suggesting incomplete convergence or metastable binding behavior. In contrast, the selected compounds satisfied all predefined statistical reliability criteria, including narrow and well-converged bootstrap confidence intervals, sufficient effective sampling sizes, and consistent per-frame energetic distributions across trajectories. Furthermore, their confidence intervals did not overlap with those of the reference inhibitor AZD9291, supporting the notion of statistically significant difference in the strength of initial, non-covalent target engagement. Collectively, these critetia identify Z5129795390, Z5129787523, and Z5129914621 as the most robust computationally predicted hit candidates, representing both energetically favorable and statistically reliable binding modes suitable for downstream experimental validation.

**Figure 4:**
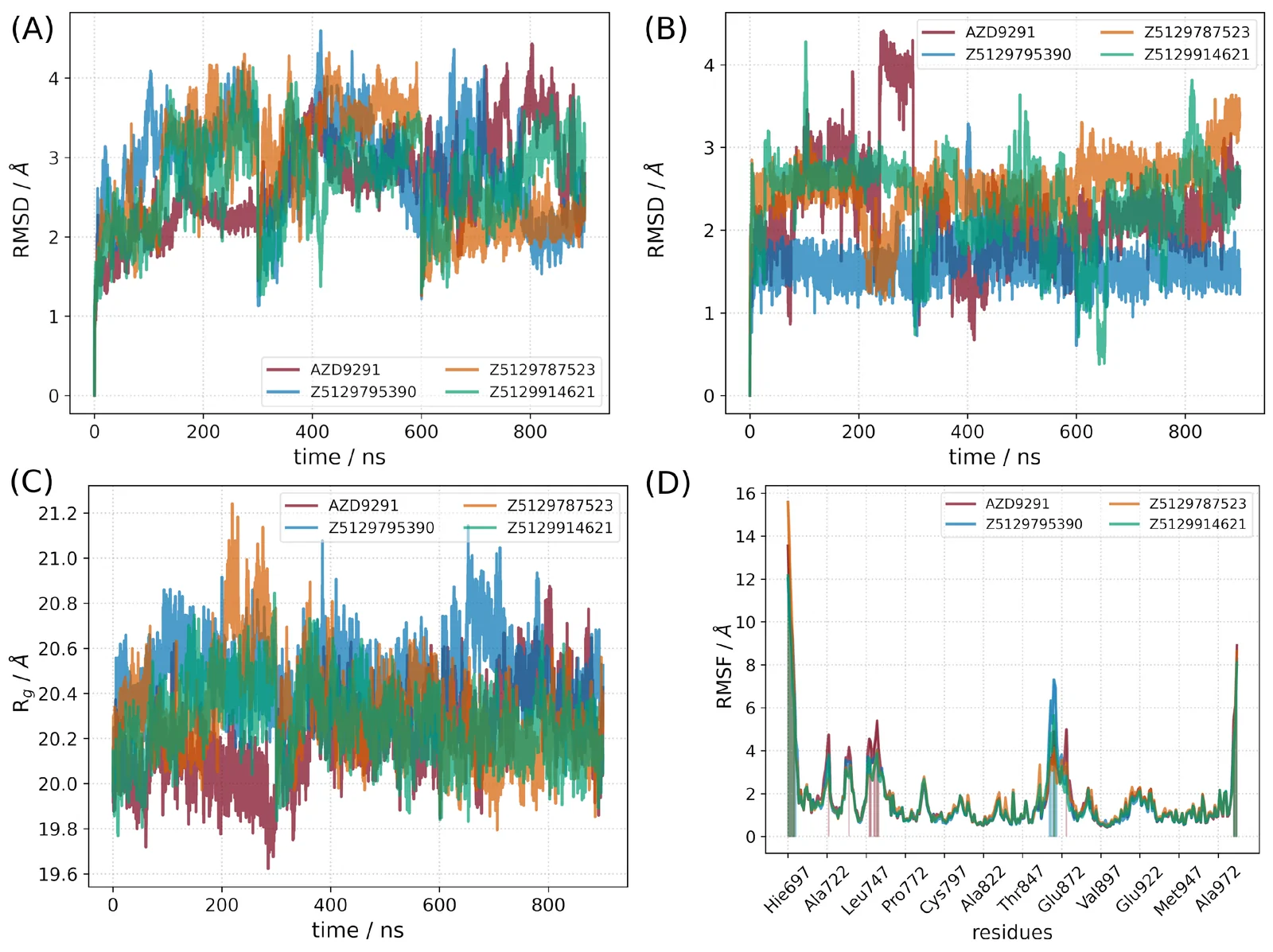
Structural stability analysis derived from 300 ns molecular dynamics simulations. Three independent MD replicates were concatenated into a single trajectory prior to analysis. (**A**) Root-mean-square deviation (RMSD) of the protein backbone atoms, (**B**) ligand RMSD relative to the initial bound conformation, (**C**) radius of gyration ($R_{g}$) of the protein, and (**D**) per-residue root-mean-square fluctuation (RMSF) calculated for heavy atoms. Amino acid residues exhibiting RMSF values exceeding 4 Å are highlighted to indicate regions of increased flexibility.

Analysis of protein backbone RMSD values indicates stable behavior across all investigated complexes following the initial equilibration phase (Figure 4A). After an expected early increase associated with structural relaxation, all systems reached a plateau characterized by moderate fluctuations around equilibrium conformations. The average RMSD values were 2.86 ± 0.61 Å for Z5129795390, 2.75 ± 0.73 Å for Z5129787523, 2.69 ± 0.53 Å for Z5129914621, and 2.67 ± 0.59 Å for AZD9291, confirming that the proposed compounds maintain overall structural stability within the binding site comparable to that of the reference inhibitor during the initial binding phase. The RMSD profiles exhibited no signatures of transient events, with values rarely exceeding 4 Å. All-atom ligand RMSD trajectories rapidly converged to stable plateaus across all complexes, confirming their persistent retention within the binding site throughout the simulations (Figure 4B). Although this behaviour was common to the entire series, the plateau heights differed markedly, reflecting ligand-specific conformational mobility. Z5129795390 exhibited the lowest plateau and the smallest standard deviation, consistent with its restricted internal flexibility of only five rotatable bonds. Conversely, AZD9291 displayed the highest plateau and the largest standard deviation, attributable to its substantially greater conformational freedom of ten rotatable bonds.

The radius of gyration ($R_{g}$) analysis further supports the formation of structurally stable complexes, showing preservation of the overall tertiary fold throughout the simulations (Figure 4C). Minor variations in $R_{g}$ values are attributed primarily to ligand-dependent adjustments in local packing rather than global structural rearrangements. Consistently, RMSF profiles calculated for heavy atoms exhibit elevated flexibility at the *N*- and *C*-terminal regions, which is characteristic for kinase domains simulated in isolation. The most flexible residues were predominantly located within terminal segments (His697–Gln701 and Gln982–Asp984), while additional localized fluctuations were ligand-dependent. For example, enhanced mobility near residues Lys754 and the αC-helix region was observed for Z5129795390 and AZD9291, whereas Z5129787523 and Z5129914621 displayed increased fluctuations of Lys867. Importantly, residues forming the catalytic core and ATP-binding site remained comparatively rigid across all systems, indicating sustained binding-site integrity during long-timescale simulations.

The remaining ligands selected for extended MD simulations displayed qualitatively similar dynamic behavior. Therefore, to avoid redundancy, their RMSD, $R_{g}$, and RMSF analyses, together with those of the apo EGFR T790M/L858R system, are presented in the Supporting Information (Figure S9), while the main text focuses on the reference inhibitor and the three highest-confidence hit compounds.

As shown in these supplementary data, the RMSD profiles of these remaining systems converge to stable plateaus after initial equilibration, with only minor ligand-dependent differences in plateau heights. Similarly, the radius of gyration and per-residue RMSF profiles remain highly consistent across all systems, confirming that the overall compactness, structural integrity, and intrinsic flexibility of the EGFR kinase domain are preserved throughout the simulations.

To further characterize the conformational state of the kinase domain, the distance between the $C_{\delta }$ atom of Glu762 (αC-helix) and the $N_{\zeta }$ atom of Lys745 (β3 strand) was monitored throughout the simulations, as this interaction represents a well-established structural marker distinguishing active (αC-in) and inactive (αC-out) EGFR conformations. Formation of the Lys745–Glu762 salt bridge stabilizes the catalytically competent active state, whereas disruption of this interaction accompanies outward displacement of the αC-helix and kinase inactivation [35,36].

The analyzed systems exhibit distinct conformational preferences reflected in the average Lys745–Glu762 distances. The Z5129914621 complex showed the shortest mean distance (6.73 ± 1.84 Å), approaching values characteristic of partially preserved or transient salt-bridge formation reported for active-like EGFR conformations (typically ∼4–6 Å) and notably shorter than the average distance observed in the apo EGFR simulation (Figure S10) [37]. In contrast, AZD9291 displayed an intermediate average separation of 9.17 ± 2.18 Å, consistent with a tendency toward an inactive or Src-like inactive kinase state conformation targeted by mutant-selective inhibitors. Larger average distances were observed for Z5129795390 (12.17 ± 2.79 Å) and Z5129787523 (9.84 ± 1.17 Å), indicating persistent disruption of the Lys745–Glu762 ionic interaction and outward positioning of the αC-helix. A clear correlation was observed between the average Lys745–Glu762 distance and the local flexibility of the αC-helix. In Figure S11, the final frame of each trajectory highlights the distance between the CD atom of Glu762 and the NZ atom of Lys745, while the per-residue RMSF is projected onto the protein backbone in a cartoon representation. Complexes exhibiting larger mean distances correspondingly display enhanced flexibility of the αC-helix, suggesting that disruption of the Lys745–Glu762 interaction contributes to increased conformational mobility within this critical secondary structure element, consistent with trends reported in the literature [15,38,39,40].

Previous molecular dynamics studies demonstrated that the Lys745–Glu762 distance increases from approximately 5–6 Å in the active kinase to values exceeding 15 Å upon transition to the inactive αC-out conformation, reflecting loss of catalytic alignment within the ATP-binding site [41]. The distance distributions observed here therefore suggest that the investigated ligands predominantly sample inactive-like conformational ensembles of the EGFR T790M/L858R mutant. Notably, the relatively shorter and less variable distance observed for Z5129914621 indicates partial sampling of active-like αC orientations, which may reflect increased conformational adaptability of the binding pocket. Such behavior is consistent with reports showing that ligand-dependent modulation of the Lys745–Glu762 interaction governs transitions between metastable kinase states and contributes to inhibitor selectivity toward mutant EGFR forms [42]. The observed conformational preferences reflect local dynamic sampling within the simulated timescale rather than complete transitions between active and inactive kinase states.

**Figure 5:**
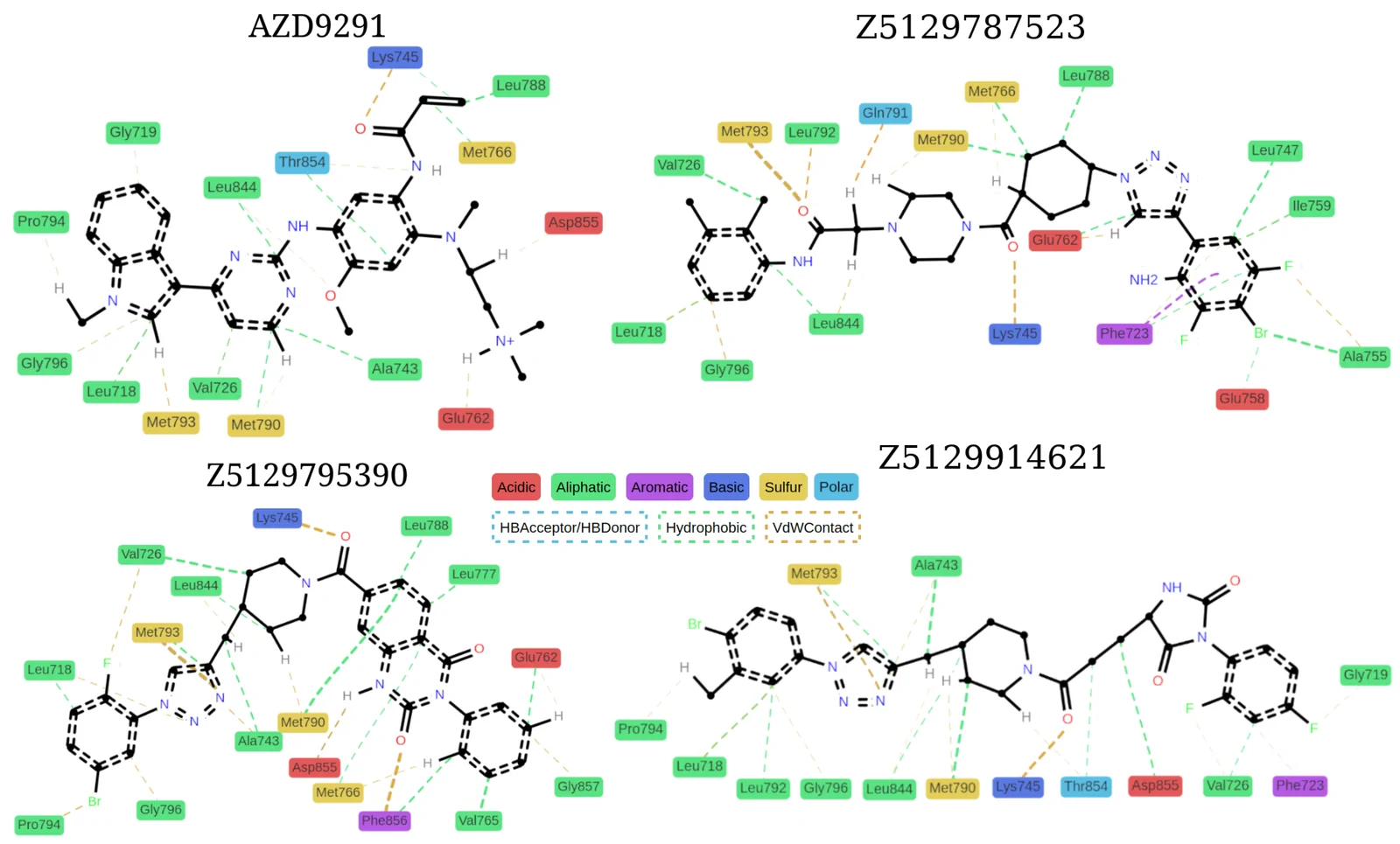
Two-dimensional interaction fingerprints for EGFR T790M/L858R complexes with AZD9291 and the top three ligands (Z5129795390, Z5129914621, Z5129787523). Only interactions present in at least 30% of the merged MD trajectory frames are shown. Line thickness is proportional to the persistence of each interaction across the trajectory.

Analysis of protein–ligand interaction fingerprints derived from ProLIF revealed a consistent interaction pattern dominated by hydrophobic contacts within the ATP-binding pocket of EGFR T790M/L858R (Figure 5). Across all investigated systems, residues lining the adenine-binding region and hydrophobic back pocket, including Leu718, Val726, Ala743, Leu788, Met766, Met790, and Leu844, exhibited the highest interaction persistence. These residues are predominantly nonpolar and form the structural core of the kinase active site, where ligand stabilization is primarily governed by hydrophobic packing and van der Waals complementarity. Such interaction profiles are consistent with crystallographic and simulation studies demonstrating that mutant-selective EGFR inhibitors rely heavily on hydrophobic engagement of the gatekeeper region surrounding Met790 to achieve both affinity and mutation selectivity [19].

The reference inhibitor AZD9291 displays a balanced interaction network combining extensive hydrophobic contacts with polar stabilization mediated by Lys745, Thr854, and Asp855. In particular, Lys745 participates in hydrogen-bond acceptor and π–cation interactions, while Asp855 contributes transient electrostatic contacts, reflecting the known role of charged residues in anchoring irreversible and mutant-selective inhibitors within the catalytic cleft [39]. This mixed interaction pattern represents a characteristic feature of clinically successful EGFR inhibitors, where hydrophobic enclosure ensures residence within the pocket while polar interactions modulate binding orientation and kinase-state stabilization.

All screened compounds largely reproduce the hydrophobic interaction framework observed for the reference ligand but additionally establish persistent hydrogen-bond interactions with Met793, a hinge-region residue critically involved in ATP recognition. The high occupancy of hydrogen-bond acceptor interactions at Met793 suggests stable hinge binding comparable to classical type I/II kinase inhibitors [43]. In the Z5129787523 complex, the 1,2,3-triazole moiety forms a hydrogen bond with Lys745, positioning the ligand within the binding site so as to prevent direct contact between Lys745 and Glu762. These interactions with Lys745 and Met793 jointly constitute the most persistent hydrogen-bonding contacts observed throughout the simulation.

A consistent relationship emerges when the machine-learning descriptors are interpreted together with the molecular dynamics analyses. SHAP identified descriptors associated with molecular hydrophobicity, aromatic nitrogen content, and spatial distribution of atomic properties as the most influential variables governing predicted activity. These physicochemical characteristics are consistent with the interaction fingerprints obtained from the production MD trajectories.

The three highest-ranked compounds maintained highly persistent hydrophobic contacts with residues lining the ATP-binding pocket, particularly Leu718, Val726, Met766, Leu788, Met790, Met793, and Leu844, which were present during the majority of the simulations. These interactions are reflected by the importance of the SlogP_VSA and VSA_EState descriptor families, indicating that an appropriate balance between hydrophobic surface area and electronic surface properties is essential for stable binding.

Similarly, the importance of aromatic nitrogen-containing fragments (SaaN) agrees with the persistent hydrogen-bond interactions observed with Met793 and, depending on the ligand, Lys745 or Thr854. MM/GBSA per-residue decomposition further confirmed that these residues contribute substantially to ligand stabilization, supporting the mechanistic interpretation of the selected descriptors.

Finally, the high ranking of topological autocorrelation descriptors (GATS4s and GATS6Z) suggests that not only the presence of specific functional groups but also their spatial arrangement contributes to activity. This interpretation is supported by the stable binding modes observed throughout the MD simulations, where the most active compounds preserved favorable interaction networks while maintaining low ligand RMSD values. Collectively, these findings indicate that the machine-learning model identifies molecular features that are consistent with the dynamic interaction patterns governing recognition of the EGFR T790M/L858R ATP-binding pocket.

## 3. Materials and Methods

### 3.1. Training of Machine Learning Model

#### 3.1.1. Dataset Preparation

The EGFR-focused ligand activity dataset was assembled from the ChEMBL database (accessed 23 June 2025) [44,45]. Specifically, data were extracted from target CHEMBL203, corresponding to a single-protein EGFR bioassay reporting experimentally determined IC_50_ values. The raw collection comprised 18,105 activity records, which were systematically curated following established best practices for bioactivity data processing [46].

Initially, records were filtered to retain only measurements reported in nanomolar units, eliminating entries with missing or non-nM standard units and yielding 16,626 data points. The dataset was then narrowed to compounds evaluated against the double-mutant EGFR (L858R/T790M) by selecting entries annotated accordingly in the “Assay Variant Mutation” field, resulting in 1720 records. Subsequently, only quantitative activity measurements with a “Standard Relation” of “=” were preserved, reducing the dataset to 1498 entries.

To resolve redundant measurements, compounds with multiple IC_50_ values were examined for consistency. Entries exhibiting variability greater than 10% were excluded, whereas measurements within this threshold were averaged to obtain a single representative value per compound, producing a dataset of 1161 records. This >10% variability threshold was used as a pragmatic criterion to identify measurements with low reproducibility or potential inconsistencies between replicates, rather than as an absolute statistical cutoff. Further standardization was achieved by removing salt forms to ensure a uniform molecular representation, leaving 1151 unique compounds. Finally, all records flagged with the comment “Outside typical range” were discarded, resulting in a curated dataset comprising 1148 molecules, which was used in all subsequent analyses.

#### 3.1.2. Molecular Descriptor Generation and Feature Selection

In order to represent the curated EGFR inhibitor set in a numerical form amenable to machine learning, an extensive collection of molecular descriptors was computed by combining the capabilities of RDKit v2024 09 5 [47] and the Mordred descriptor framework [48]. As a first step, SMILES strings were transformed into three-dimensional molecular structures using RDKit’s conformer generation algorithms. For each compound, a set of five conformations was generated and subsequently optimized using the MMFF94 molecular mechanics force field implemented in RDKit [49]. From this ensemble, the minimum-energy conformer was selected as the canonical geometry and saved in SDF format for further processing.

Descriptor calculation was carried out with Mordred, which provides a comprehensive range of one-dimensional (1D), two-dimensional (2D), and three-dimensional (3D) molecular descriptors. While 1D and 2D descriptors encode constitutional, topological, and physicochemical properties independent of molecular conformation, 3D descriptors were derived directly from the MMFF94-optimized geometries to ensure a consistent structural basis. Following descriptor generation, all features containing missing values or non-numerical entries were removed, resulting in a fully numeric descriptor matrix comprising 1649 descriptors suitable for subsequent modeling steps.

To reduce dimensionality and eliminate redundant or uninformative features, a hierarchical feature filtering strategy was employed. First, descriptors dominated by zero values were excluded by removing those with more than 85% zero entries, thereby mitigating sparsity-related effects. Second, descriptors exhibiting minimal variation across the dataset were discarded by applying a variance threshold, excluding features with a standard deviation below 3%. In the final filtering stage, pairwise Spearman correlation coefficients were computed to identify highly collinear descriptors. For descriptor pairs with correlation coefficients exceeding 0.9, the feature showing a weaker absolute correlation with the experimental pIC_50_ values was removed. This sequential filtering procedure reduced the descriptor space to 393 chemically interpretable features.

Prior to feature ranking and model construction, all retained molecular descriptors were standardized to zero mean and unit variance using the StandardScaler transformation, ensuring comparability across features with differing numerical ranges. This normalization step was applied to prevent scale-dependent bias in distance-based algorithms and to facilitate stable model training.

A subsequent feature prioritization step was performed to identify the most informative descriptors for regression modeling. For this purpose, the RReliefF algorithm [50], an extension of the classical ReliefF approach designed for continuous response variables, was employed. RReliefF quantifies feature relevance by assessing how variations in individual descriptors correspond to changes in biological activity among neighboring compounds in descriptor space. The algorithm was applied within a five-fold resampling scheme, and feature importance scores were aggregated across folds to enhance robustness and reduce sampling variance. Based on the resulting rankings, the top 50 consistently informative descriptors were selected and retained for model development.

Using the hyperparameters optimized via Optuna [51], the dimensionality of the feature space was further reduced through a wrapper-based selection strategy. Specifically, a sequential feature selector was employed to iteratively identify a compact subset of descriptors that maximized predictive performance. SFS operates by progressively adding or removing features based on their marginal contribution to a predefined objective function, allowing interactions between descriptors to be implicitly accounted for during selection. This approach enables the identification of feature subsets that are optimally tailored to the learning algorithm, rather than relying solely on univariate relevance criteria [52].

Starting from the Optuna-optimized baseline model, the SFS procedure was applied to systematically decrease the number of input descriptors. This iterative refinement resulted in a final subset of 16 features, which provided the best trade-off between model complexity and predictive accuracy. Using this reduced descriptor set, a second round of hyperparameter optimization was conducted independently for three regression algorithms: XGBoost [53], support vector machines [54], and random forests [55]. For each model class, Optuna was used to re-optimize algorithm-specific hyperparameters to ensure that performance comparisons were not biased by suboptimal tuning.

Following this final optimization stage, regression models based on XGBoost, SVM, and RF were trained using the selected 16-feature representation. These optimized models constituted the final predictive frameworks evaluated in the subsequent analysis.

### 3.2. Virtual Screening and Applicability Domain Assessment

A virtual screening campaign was conducted on a selected portion of the Enamine REAL chemical space [56]. To guarantee full compatibility with the trained predictive framework, all screened compounds were processed through an identical computational pipeline as the training molecules. In this workflow, SMILES representations were first transformed into three-dimensional molecular structures, followed by the generation and MMFF94-based optimization of multiple conformers. Molecular descriptors were then calculated, after which only the descriptors retained during model development were extracted. As a result, activity predictions were generated strictly within the descriptor space used to train the XGBoost regression model.

Before virtual screening, the applicability domain (AD) of the final XGBoost model was assessed using the Mahalanobis distance computed in the original 16-dimensional descriptor space. Unlike projection-based approaches, the Mahalanobis distance accounts for the covariance structure of the descriptor matrix and therefore provides a multivariate measure of similarity between screened compounds and the chemical space represented by the training set [57,58,59].

The centroid of the training set was calculated as the mean descriptor vector, while the covariance matrix was estimated from the training descriptors. To ensure numerical stability during matrix inversion, a small diagonal regularization term ($10^{-5}$) was added to the covariance matrix. The Mahalanobis distance of each screened compound from the training-set centroid was then computed as

(2) $$D_{M}\left(x\right)=\sqrt{{\left(x-\mu \right)}^{T}\Sigma^{-1}\left(x-\mu \right)},$$

where x is the descriptor vector of the screened compound, μ is the centroid of the training set, and $\Sigma^{-1}$ denotes the inverse covariance matrix.

Compounds were considered to lie within the applicability domain when their Mahalanobis distance did not exceed the statistical threshold corresponding to the 95% confidence region of the multivariate normal distribution,

(3) $$D_{M}\leq \sqrt{\chi_{p,\;0.95}^{2}},$$

where *p* is the number of descriptors retained in the final model (p=16). Compounds exceeding this threshold were classified as outside the applicability domain and excluded from subsequent virtual screening analyses.

For visualization purposes only, both the training compounds and screened molecules were projected onto the first two principal components using principal component analysis (PCA). The resulting two-dimensional representation was used solely to illustrate the distribution of compounds classified as inside or outside the applicability domain and did not influence the applicability domain assignment itself.

A PAINS filtering step was subsequently applied to the top 100 machine learning-ranked compounds to eliminate structures associated with potential assay interference and frequent hitters. Five compounds were identified as PAINS and excluded from further analysis. The remaining highest-ranked PAINS-free compounds were prioritized, and the top 50 were advanced to the structure-based screening workflow comprising molecular docking, molecular dynamics simulations, and MM/GBSA binding free energy calculations.

### 3.3. Molecular Docking

The atomic coordinates of the human epidermal growth factor receptor (EGFR) were retrieved from the Protein Data Bank (PDB ID: 3W2P), representing the kinase domain crystallized in complex with *N*-2-[4-(3-chloro-4-[3-(trifluoromethyl)phenoxy]phenylamino)-5*H*-pyrrolo[3,2-*d*]pyrimidin-5-yl]ethyl-4-(dimethylamino)butanamide (W2P) [60,61]. Prior to further processing, all crystallographic water molecules and the co-crystallized ligand were removed. The receptor model was truncated to include residues ranging from His697 to Asp984, encompassing the catalytically relevant kinase region.

Protonation states of titratable amino acid side chains were assigned using the PROPKA server [62], which estimates residue-specific p$K_{a}$ values and determines the dominant protonation state at physiological pH (pH 7.4). The resulting protonated receptor structure served as the common starting point for both molecular docking calculations and subsequent molecular dynamics simulations in apo and ligand-bound forms.

For docking preparation, the receptor was processed using the mk_prepare_receptor.py script, generating a PDBQT representation with polar hydrogens retained and Gasteiger partial charges assigned. Ligand structures were prepared using the mk_prepare_ligand.py pipeline developed by the Forli Lab (CCSB) [63]. Three-dimensional ligand geometries were constructed from SMILES strings, after which ten conformers per compound were generated and optimized using the MMFF94 force field as implemented in RDKit. The minimum-energy conformer for each ligand was selected and converted to PDBQT format. Docking calculations were restricted to the top 50 PAINS-free compounds prioritized by the trained XGBoost regression model.

The docking search region was defined based on the binding site of W2P in the crystal structure. Specifically, the center of mass of the co-crystallized inhibitor (coordinates: x=145.32, y=24.69, z=49.75 Å) was used to define the grid center, around which a cubic search box of dimensions 25×25×25 Å^3^ was constructed. Docking simulations were carried out using AutoDock Vina 1.2.6 with an exhaustiveness parameter of 100, num_modes set to 100, and an energy range of 4 kcal mol^−1^, ensuring extensive sampling of ligand orientations and conformations [64].

Upon the completion of the docking calculations, ligand poses were ranked according to AutoDock Vina’s scoring function. High-ranking poses were subsequently subjected to visual inspection to verify chemically and structurally plausible binding modes, characterized by appropriate steric accommodation within the ATP-binding cleft and the formation of reasonable hydrogen-bonding and hydrophobic interaction patterns. Docked complexes satisfying these criteria were selected as initial configurations for molecular dynamics simulations, thereby ensuring that downstream dynamical analyses were initiated from physically meaningful EGFR–ligand binding geometries.

To validate the molecular docking protocol, the co-crystallized ligand W2P was redocked into the EGFR T790M/L858R crystal structure using the same AutoDock Vina settings employed throughout this study. Because W2P is covalently linked to Cys797 in the crystal structure, whereas the present workflow used a non-covalent representation to generate initial structures for molecular dynamics simulations, docking accuracy was assessed using both the overall ligand RMSD and the heavy-atom RMSD of the rigid binding core. The heavy-atom RMSD of the rigid core was 0.64 Å, demonstrating excellent reproduction of the crystallographic binding mode (Figure S1). The larger overall RMSD (5.53 Å) originated predominantly from the flexible ethyl-4-(dimethylamino)butanamide substituent, which is conformationally unrestricted in the absence of the covalent Cys797–ligand bond.

### 3.4. Molecular Dynamics Simulations

All the molecular dynamics simulations were carried out using the AMBER22 package on NVIDIA A100 GPU accelerators provided by the Supek HPC infrastructure [65]. Two types of systems were analyzed in this study: the unliganded (apo) EGFR kinase domain and the protein–ligand complexes with selected candidate inhibitors. An identical simulation protocol was used for these two types of systems to ensure methodological comparability and to enable the comparison of the dynamic behavior of these systems.

The apo protein system was built upon the carefully curated crystal structure of EGFR (PDB ID: 3W2P). To focus on the catalytically relevant kinase domain of EGFR while excluding non-essential regions, the protein residues from His697 to Asp984 were retained in the apo protein system. The protonation state of titratable residues was computed using the PROPKA method under physiological pH conditions to ensure realistic electrostatics of the system before further preparation and simulation. In the protein–ligand systems, the prepared protein structure was merged with the binding poses of ligands obtained from the docking simulations using the AutoDock Vina method. These complexes were subjected to the same parametrization, equilibration, and production protocols as the apo system, thereby enabling a controlled assessment of ligand-induced conformational effects. These protein–ligand complexes were then treated the same as the apo system, as described previously, to assess the effect of the ligands on the system.

The protein atoms were described using the ff19SB force field [66], while the solvent was treated explicitly using the OPC water model [67]. The ligand topology and parameters were generated using the antechamber and parmchk2 utilities and GAFF2 force field [68]. The partial charges for the ligands were determined using the semiempirical AM1-BCC method, which strikes the right balance for the electrostatics required for the simulation. Halogen-specific anisotropic effects (e.g., sigma–hole interactions) are not explicitly represented in the employed force field and are therefore not quantitatively resolved in this study.

Prior to dynamic propagation, each system underwent a multi-stage energy minimization protocol to remove unfavorable contacts and relax the solvent environment. In the initial stage, all protein atoms were restrained with a strong harmonic potential (999.0 kcal mol^−1^ Å^−2^), and 60,000 minimization steps were performed (30,000 steepest descent followed by 30,000 conjugate gradient). In the second stage, restraints were reduced and applied only to backbone atoms (50.0 kcal mol^−1^ Å^−2^), followed by 40,000 additional steps (20,000 steepest descent and 20,000 conjugate gradient). Finally, a fully unrestrained minimization of 30,000 steps was carried out to allow complete relaxation of all atomic degrees of freedom before thermal equilibration.

After minimization, the systems were heated gradually from 0 K to 310 K under constant volume conditions (NVT). The heating process occurred in five segments of 50,000 steps with 2 fs in each step. The temperature control was achieved with a Langevin thermostat with a frequency of 1.0 ps^−1^. The covalent bonds of all hydrogen atoms were constrained with SHAKE to allow stable integration with this time step [69].

The equilibration phase followed in the isothermal–isobaric (NPT) ensemble at a temperature of 310 K for a period of 250,000 steps, which corresponds to a time of 0.5 ns and a time step of 2 fs. The simulations were performed under isotropic pressure scaling in the presence of periodic boundary conditions. The simulations also incorporated a relaxation time of 2 ps. The simulations were maintained at a constant temperature by a Langevin thermostat with a coupling constant γ=1.0 ps^−1^. The simulations incorporated a cutoff of 10 Å for short-range interactions. The simulations also incorporated the use of the particle mesh Ewald (PME) method for long-range electrostatic interactions [70]. The SHAKE restraints on bonds containing hydrogen atoms were also maintained.

For the production simulations of the complexes, the simulations were carried out in the NPT ensemble with a constant temperature of 310 K and used a 2 fs time step. The simulations were controlled using a Langevin thermostat with γ=1.0 ps^−1^ and an isotropic pressure coupling with $\tau_{p}=2$ ps. The short-range nonbonded cut-off was set to 10 Å, and the long-range electrostatics were handled using the PME method. In addition, SHAKE constraints were used on all bonds containing a hydrogen atom. The coordinates, velocities, and energy components were saved every 100,000 time steps.

From the PAINS-cleared complexes with the highest ML scores, the top 50 were selected for 50 ns simulations to enable faster post-docking refinement and binding free energy estimations. Finally, the top nine complexes were selected for further simulations. In these simulations, three independent simulations of 300 ns each were run on each of these complexes. In each of these simulations, the initial velocity distribution was sampled from a Maxwell–Boltzmann distribution.

### 3.5. Binding Free Energy Calculations

Binding affinities of EGFR toward the prioritized ligands were estimated using the molecular mechanics/generalized Born surface area (MM/GBSA) approach as implemented in the MMPBSA.py program of the AmberTools package [71]. A single-trajectory protocol was employed, whereby all energetic components for the complex, receptor, and ligand were extracted from the same MD trajectory of the bound state. This strategy reduces statistical noise associated with structural inconsistencies that can arise when separate simulations are used for the individual components.

The binding free energy was evaluated according to

(4) $$\Delta G_{\mathrm{bind}}=\Delta E_{\mathrm{MM}}+\Delta G_{\mathrm{sol}}-T\Delta S,$$

where the molecular mechanics term is decomposed as

(5) $$\Delta E_{\mathrm{MM}}=\Delta E_{\mathrm{bonded}}+\Delta E_{\mathrm{elec}}+\Delta E_{\mathrm{vdW}}.$$

Here, $\Delta E_{\mathrm{bonded}}$ comprises bond, angle, and dihedral contributions, while $\Delta E_{\mathrm{elec}}$ and $\Delta E_{\mathrm{vdW}}$ correspond to electrostatic and van der Waals interactions in the gas phase, respectively. The solvation free energy is expressed as

(6) $$\Delta G_{\mathrm{sol}}=\Delta G_{\mathrm{polar}}+\Delta G_{\mathrm{nonpolar}},$$

with the polar component computed using the generalized Born implicit solvent model and the nonpolar contribution estimated as a linear function of the solvent-accessible surface area. In the present study, the entropic contribution (TΔS) was neglected, and $\Delta G_{\mathrm{bind}}$ therefore reflects the enthalpic component of binding within the MM/GBSA framework.

### 3.6. Replica Reproducibility and Hit Selection

Due to the intrinsic temporal correlation of MD trajectories, successive frames cannot be treated as statistically independent [72,73,74]. To address this, the autocorrelation function of each ligand trajectory was computed to estimate the correlation time, τ, defined as

$$\tau =1+2\sum_{k=1}^{M}\rho \left(k\right),$$

where ρ(k) is the autocorrelation at lag *k* and *M* is the maximum lag considered. Effective independent frames were obtained by subsampling every ⌈τ⌉ frames from the original trajectory. This procedure ensures that subsequent statistical analyses are based on effectively independent samples, mitigating artificial inflation of confidence intervals.

For each ligand, the mean binding free energy ($\Delta G_{\mathrm{bind}}$) was estimated using bootstrap resampling of the independent frames. A total of 5000 bootstrap replicates were generated, and the 2.5% and 97.5% quantiles were used to construct 95% confidence intervals. Bootstrap statistics were reported alongside the effective sample size ($N_{\mathrm{eff}}$) to provide a robust measure of uncertainty.

Reproducibility across the three independent replicates was assessed by computing the mean $\Delta G_{\mathrm{bind}}$ per replicate and its standard deviation across replicates. Additionally, the average per-frame fluctuation within each replicate was computed to quantify trajectory stability. Ligands were classified as variable if the inter-replica standard deviation exceeded 5 kcal/mol or if the average frame-level fluctuation exceeded 15 kcal/mol.

To identify hit candidates, ligands were ranked based on the mean bootstrap $\Delta G_{\mathrm{total}}$, adjusted for inter-replica variability and per-frame fluctuations.

## 4. Study Limitations

Several factors that are currently inherent to the computational workflow should be kept in mind while interpreting the findings. Firstly, the performance of the machine learning models is heavily dependent on the chemical diversity and quality of the training set compounds. Therefore, while interpreting the findings, it should be kept in mind that there may be limitations in extrapolating to structurally dissimilar compounds, even if their applicability is checked.

Secondly, the covalent inhibitor osimertinib was modelled using a non-covalent molecular dynamics and MM/GBSA framework. In this work, osimertinib is represented only in its initial non-covalent binding pose within the EGFR active site, without explicit modelling of the subsequent covalent bond formation with Cys797. Consequently, the calculated interaction energies do not capture the full thermodynamic contribution of covalent inhibition. Nevertheless, this approximation allows consistent comparative analysis across all ligands under a unified non-covalent protocol and provides insight into pre-reactive binding modes relevant for target engagement.

Third, the docking and subsequent estimation of the binding free energy using the MM/GBSA method are based on approximate calculations and implicit solvation models, which do not account for all the entropic contributions and long-range timescale movements that can affect the binding affinities. Consequently, the calculated MM/GBSA binding energies should be interpreted primarily as relative estimates for comparative ligand ranking rather than as absolute binding free energies. While the binding affinities may be approximated using molecular dynamics simulations, it should be kept in mind that there are practical limitations to the timescale that can be explored using this approach. While running the simulations in triplicate helps in obtaining more accurate statistical sampling, there can be inherent biases in the starting structures that can affect the final outcomes.

In addition to this, there are other factors that can affect the final outcomes, such as the parameterization of the ligands in the molecular mechanics calculations and the handling of long-range electrostatics, which can affect the final outcomes in terms of the persistency of the interactions and the appearance of stable structures in the protein–ligand complex. Furthermore, tautomeric forms were not explicitly enumerated or standardized, which may introduce additional structural uncertainty in ligand representation. It should be kept in mind that while this approach can predict the inhibitory activity, this cannot be validated until experimental findings are taken into account in subsequent studies.

A recognized limitation of the initial high-throughput virtual screening phase is the reliance on vendor-predefined protonation states and tautomers from the Enamine database. While Enamine prepares libraries using industry-standard protocols optimized for physiological pH (pH 7.4), the lack of exhaustive in-house tautomer enumeration and standardization represents a trade-off between computational feasibility and chemical space coverage. Consequently, certain rare tautomers or specific protonation states stabilized exclusively by the asymmetric microenvironment of the EGFR double-mutant binding pocket might be underrepresented. This could potentially lead to the omission of alternative binding modes or false negatives, a factor that should be carefully considered in downstream hit optimization.

## 5. Conclusions

In this study, we have implemented a hybrid computational approach that combines machine learning-based prioritization with structure-based molecular modeling to identify and prioritize candidate ligands targeting the EGFR T790M/L858R mutant. The application of predictive modeling, molecular docking, molecular dynamics simulations, and MM/GBSA calculations enabled us to progressively narrow down potential compounds and account for the behavior of the complex under dynamic conditions.

The short timescale of molecular dynamics simulations was used as a rapid approach to narrow down and rank potential inhibitors, and further long timescale simulations were carried out to gain insight into structural stability and the persistence of interactions in the kinase binding site. Additional simulations of the apo EGFR mutant and its complex with the reference inhibitor AZD9291 were also performed to establish a structural baseline for non-covalent binding-site interactions, providing a comparative context for our computationally prioritized candidates which now await experimental validation.

The structural features of potential inhibitors, including the stability of the protein backbone, the propensity of a compound to assume a consistent conformation upon binding, the flexibility of residues in the complex, and key intra-residue distances associated with kinase activation, were examined and found to suggest that selected compounds are likely to drive the EGFR T790M/L858R mutant into inactive states that are stabilized by inhibitor binding. The calculated MM/GBSA binding energies are consistent with our findings and suggest that this approach is likely reliable as it is consistent with dynamic behavior.

This computational approach provides a framework for effectively narrowing down potential inhibitors of the EGFR T790M/L858R mutant and gaining insight into dynamic behavior and provides a rationale for potential inhibitors of this cancer-causing kinase.

## Figures and Tables

**Table 1:** Summary of predictive performance for XGBoost, Random Forest (RF), and Support Vector Machine (SVM). Metrics are reported as mean ± standard deviation for cross-validation (CV) folds and as single values for the independent validation set. PCC = Pearson correlation coefficient.

Dataset	Model	RMSE	MAE	PCC
CV (train folds)	XGBoost	0.608±0.006	0.482±0.004	0.873
	RF	0.715±0.006	0.567±0.006	0.846
	SVM	0.743±0.011	0.557±0.007	0.784
CV (test folds)	XGBoost	0.868±0.035	0.681±0.027	0.667
	RF	0.904±0.032	0.717±0.025	0.654
	SVM	0.911±0.056	0.702±0.038	0.621
Full training set	XGBoost	0.617	0.490	0.867
	RF	0.706	0.560	0.847
	SVM	0.741	0.555	0.782
Independent validation	XGBoost	0.830	0.651	0.682
	RF	0.848	0.673	0.691
	SVM	0.842	0.647	0.674

## Data Availability

The AMBER trajectories of the EGFR–ligand complexes are openly available in the FULIR repository at https://data.fulir.irb.hr/object/irb:868 (accessed on 17 March 2026).

## Supplementary Materials

The following supporting information can be downloaded at https://www.mdpi.com/article/10.3390/ijms27167122/s1.

## Abbreviations

The following abbreviations are used in this manuscript:

AD	applicability domain
AZD9291	osimertinib
EGFR	epidermal growth factor receptor
MD	molecular dynamics
ML	machine learning
MM/GBSA	molecular mechanics/generalized Born surface area
PCA	principal component analysis
PME	particle mesh Ewald
RF	random forest
$R_{g}$	radius of gyration
RMSD	root-mean-square deviation
RMSE	root-mean-square error
RMSF	root-mean-square fluctuation
SBVS	structure-based virtual screening
SFS	sequential feature selection
SVM	support vector machines
XGBoost	extreme gradient boosting
