# Supplementary material for: From Descriptor Learning to Binding Stability: An Explainable Machine Learning Pipeline for EGFR Double-Mutant Inhibitor Discovery
Source: Int J Mol Sci. 2026 Aug 8;27(16):7122. doi: 10.3390/ijms27167122 (PMC13513548; doi:10.3390/ijms27167122)
Supplement: Supplementary file 1 [file ijms-27-07122-s001.zip › ijms-4413476-supplementary.pdf]

Supplementary Materials: From Descriptor Learning to Binding Stability: An Explainable Machine Learning Pipeline for EGFR Double-Mutant Inhibitor Discovery

Jurica Novak [1]

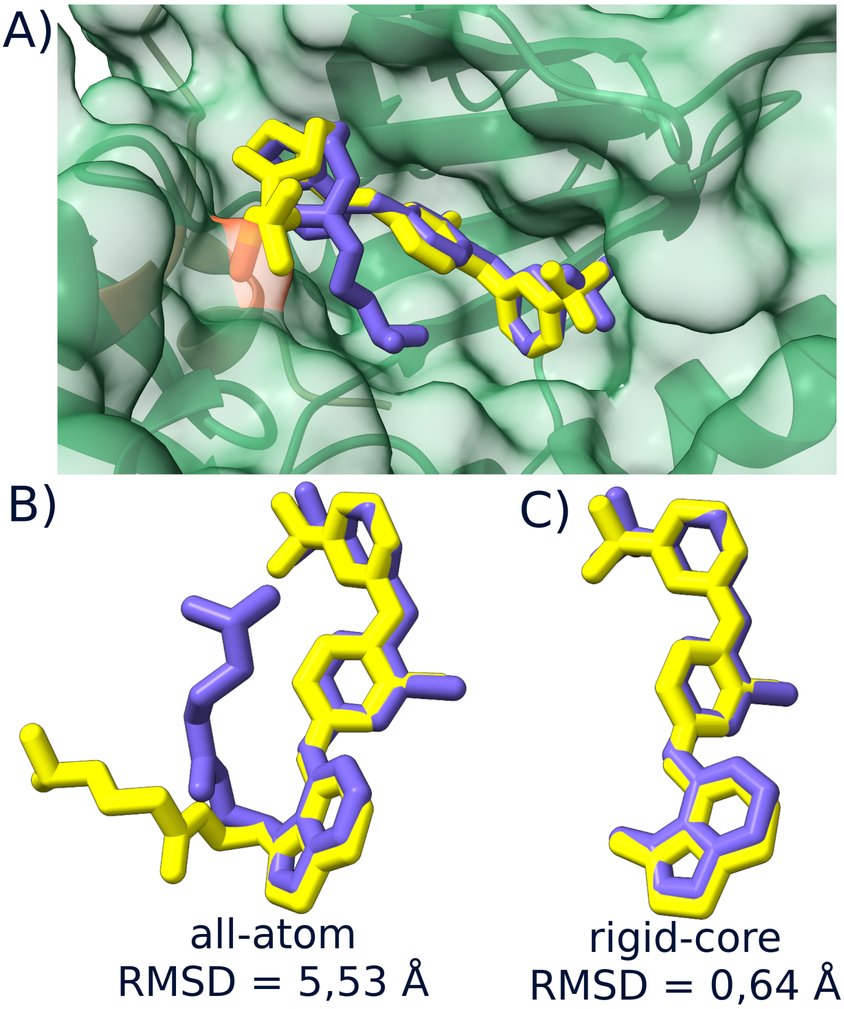

Figure S1. Validation of the molecular docking protocol by redocking the co-crystallized ligand W2P into the EGFR T790M/L858R crystal structure using the same non-covalent AutoDock Vina protocol employed to generate the initial molecular dynamics structures. **(A)** Superposition of the crystallographic ligand (yellow) and the best-scoring docked pose (blue) within the ATP-binding site. The protein is shown as a green cartoon, while the side chain of Cys797 is highlighted in orange to emphasize the position of the covalent attachment in the crystal structure. **(B)** Overlay of the complete crystallographic and docked ligand structures. The overall ligand RMSD was 5.53 Å, with the deviation originating predominantly from the flexible ethyl-4-(dimethylamino)butanamide moiety, which is covalently linked to Cys797 in the crystal structure but remains unconstrained in the non-covalent docking protocol. **(C)** Overlay of the rigid ligand core (*N*-{2-[4-({3-chloro-4-[3-(trifluoromethyl)phenoxy]phenyl}amino)-5*H*-pyrrolo[3,2-*d*]pyrimidin-5-yl]}) showing excellent agreement between the crystallographic and docked conformations, with a heavy-atom RMSD of 0.64 Å.

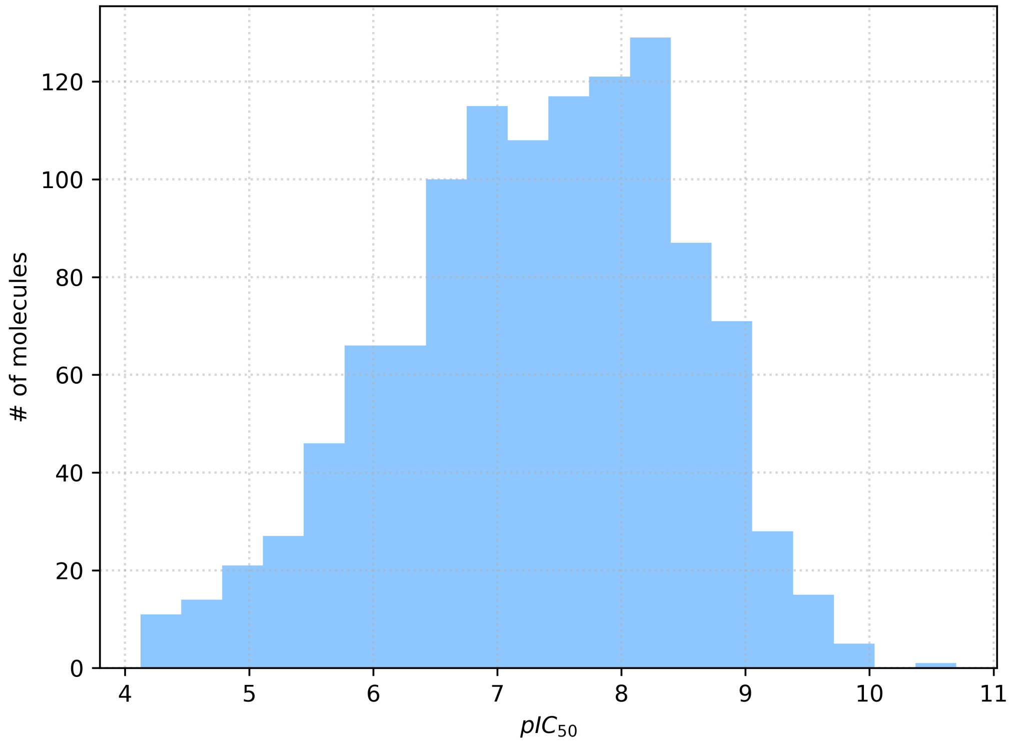

Figure S2. Distribution of experimental pIC_{50} values values for the complete curated dataset containing 1,148 unique molecules. The histogram illustrates the activity landscape of the dataset used for machine learning model development, highlighting the overall range and density of inhibitory activities that define the chemical space explored during model training and validation.

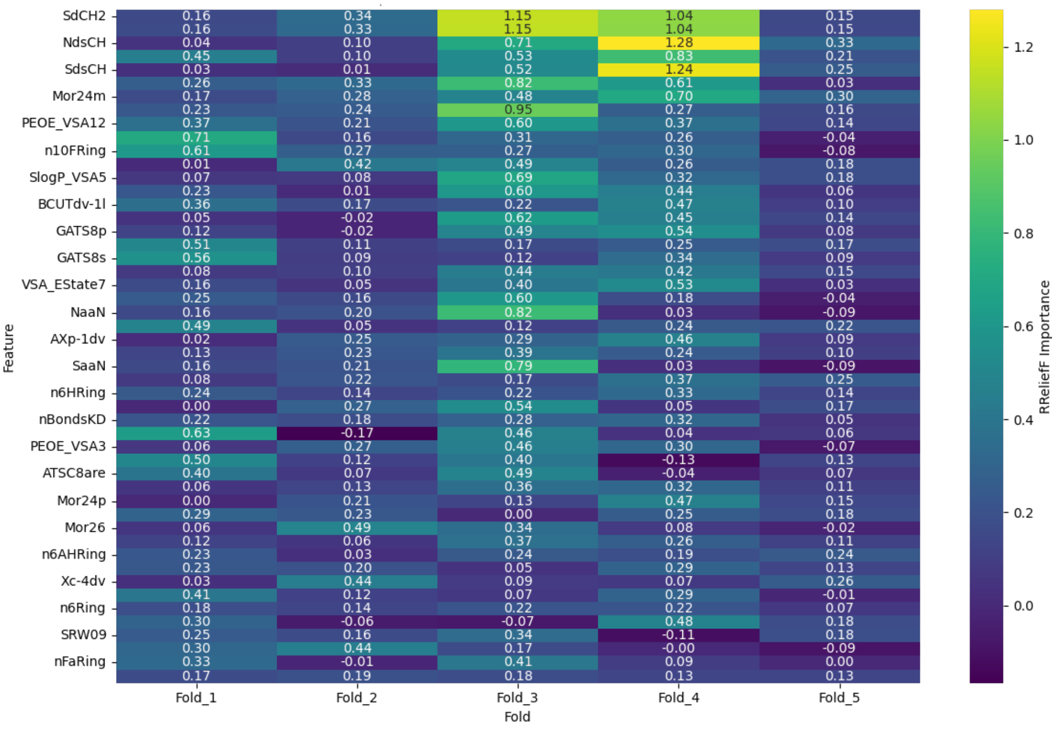

Figure S3. Heatmap illustrating the relative importance of the top 25 molecular descriptors identified by the RReliefF feature selection procedure. Descriptor relevance was evaluated within each cross-validation fold using an XGBoost regression model configured with the optimized hyperparameter set obtained through Optuna optimization.

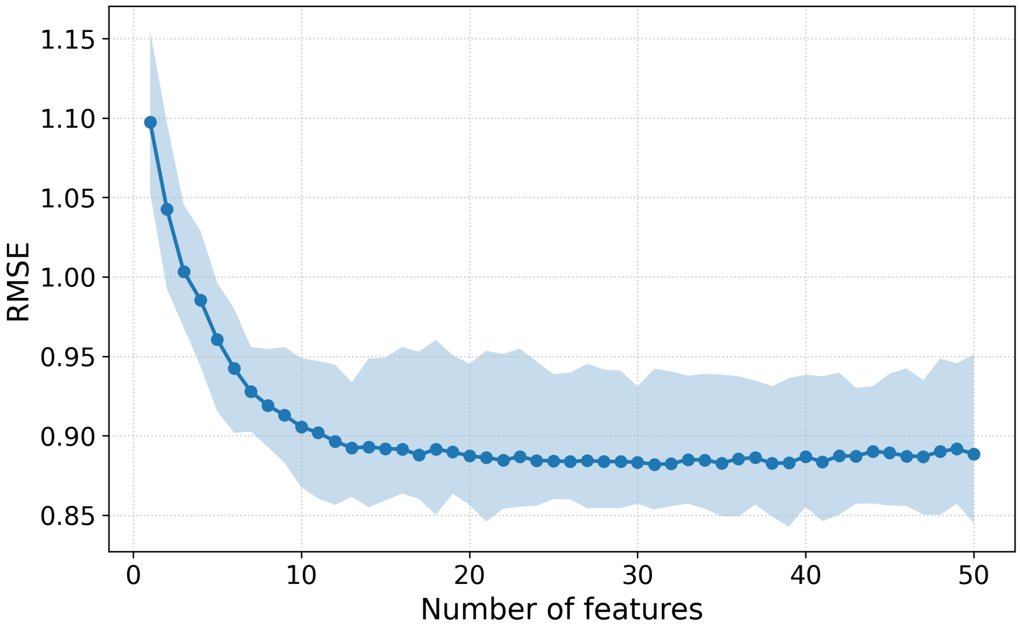

Figure S4. Dependence of XGBoost model performance on the number of retained descriptors during sequential feature selection. The plot shows the cross-validated RMSE as a function of the number of selected features. Shaded regions represent the corresponding 95% confidence intervals derived from cross-validation folds.

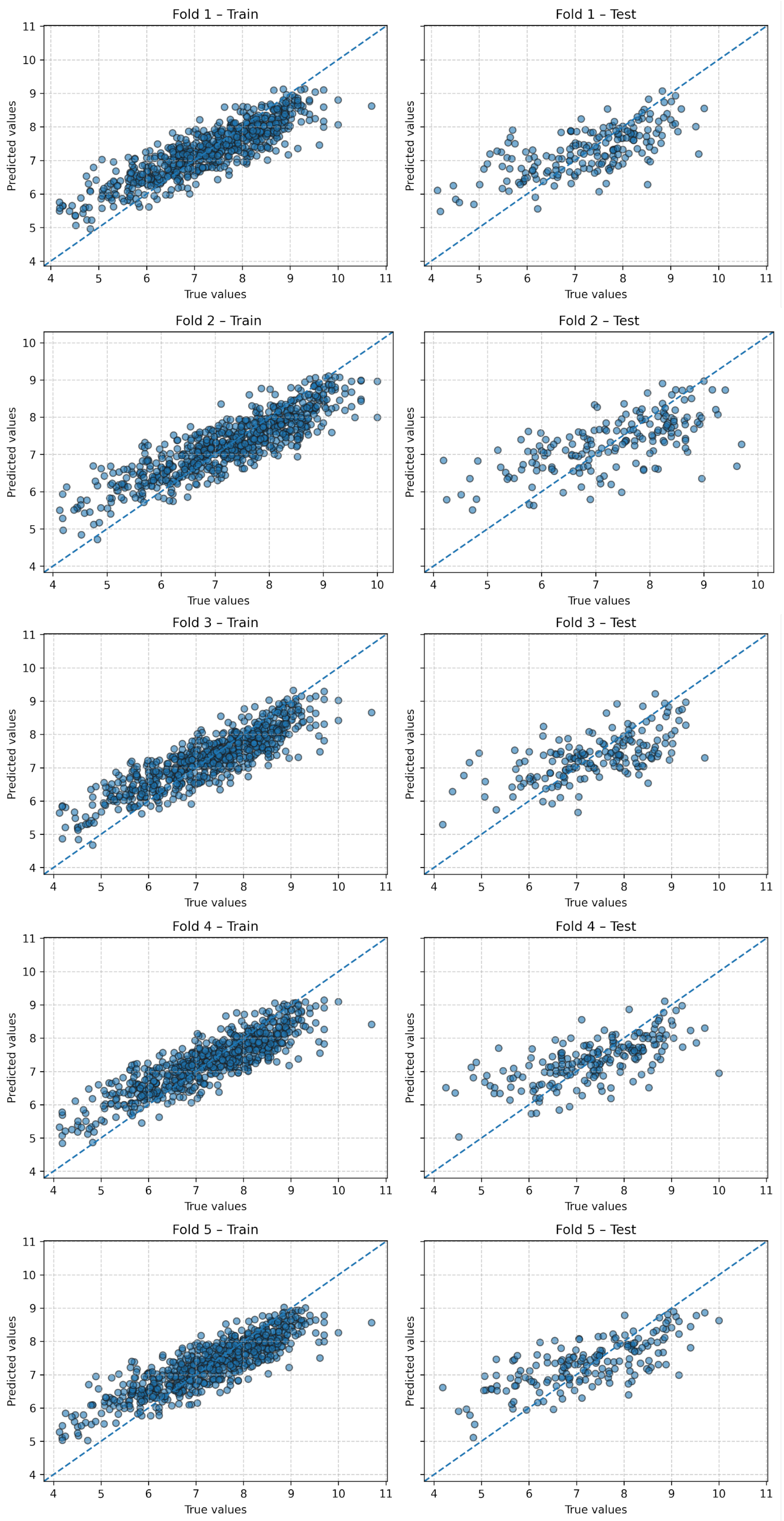

Figure S5. True versus predicted pIC_{50} values for the XGBoost model across all five scaffold-aware cross-validation folds. Each row corresponds to one fold, with training set predictions shown on the left and test set predictions on the right.

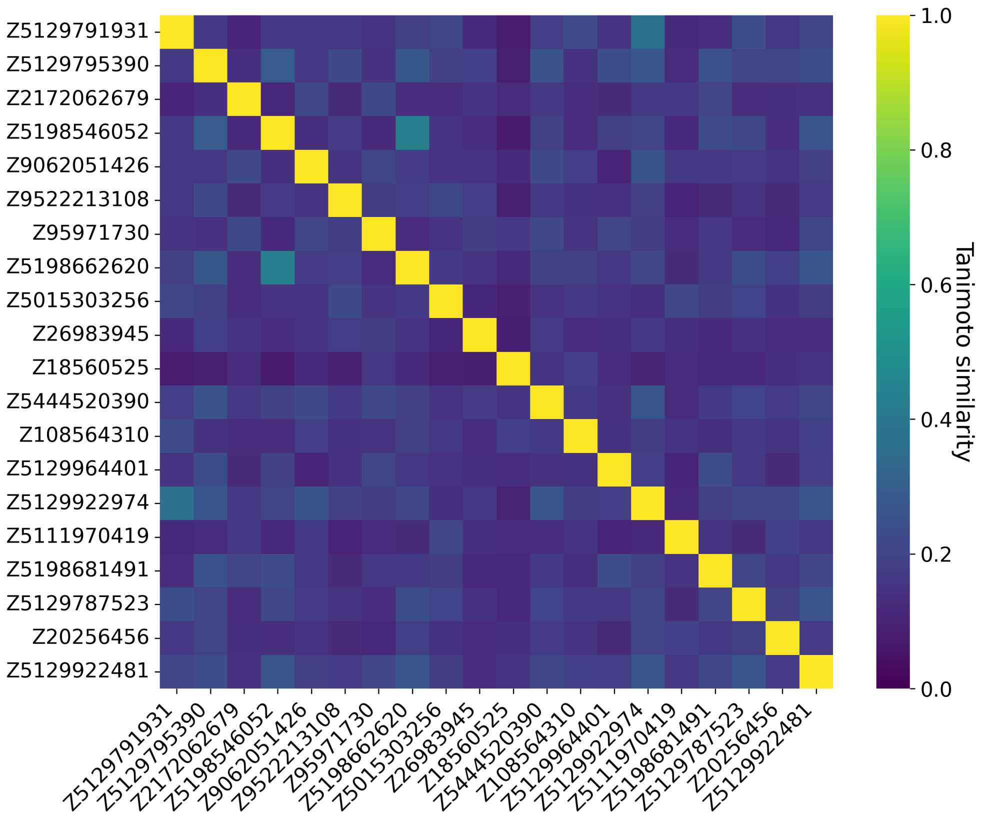

Figure S6. Pairwise Tanimoto similarity heatmap for the 20 top-ranked compounds with the highest predicted pIC_{50} values. Molecular fingerprints were generated using the Morgan algorithm (radius = 2, fingerprint length = 2048).

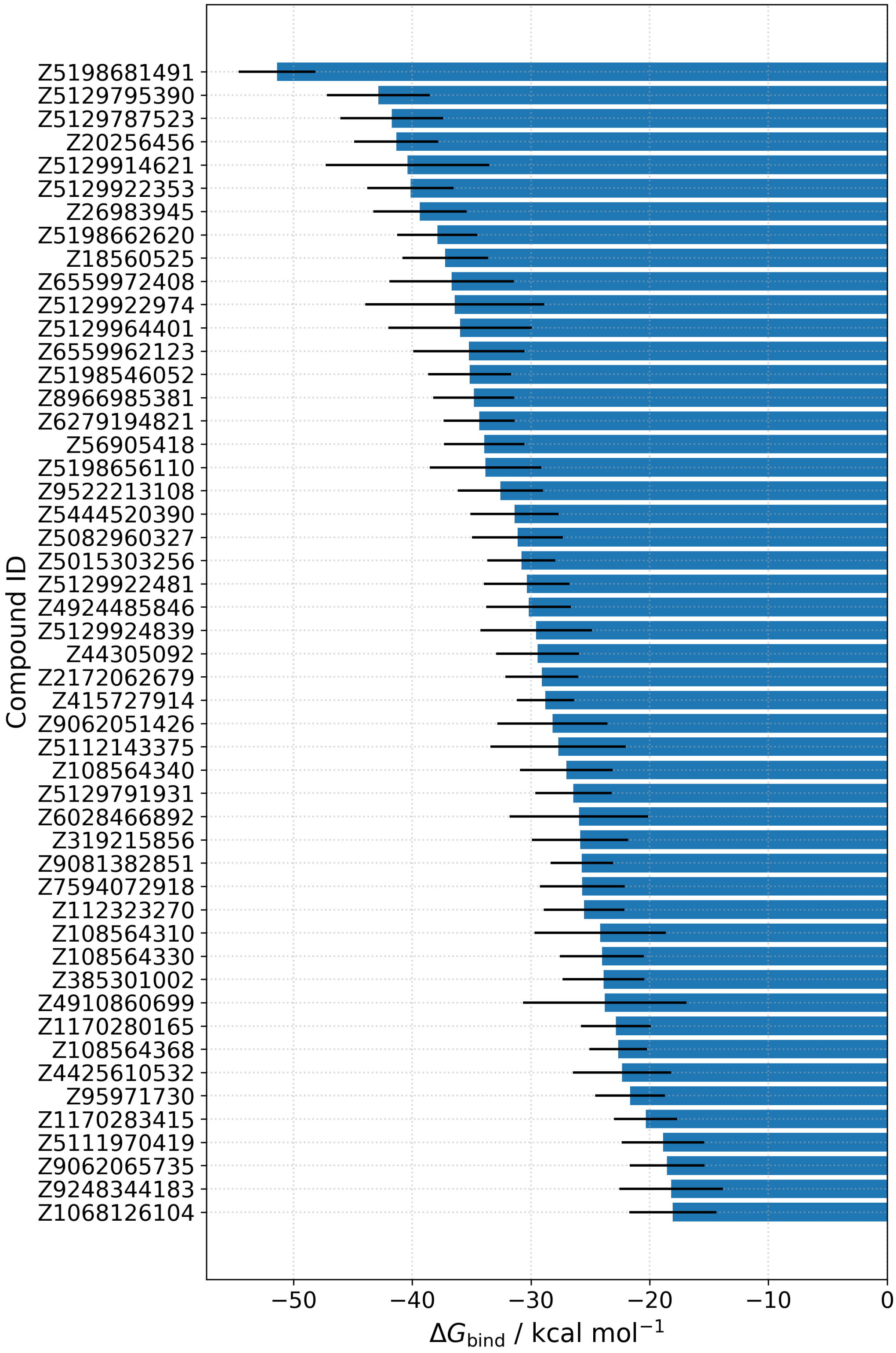

Figure S7. MM/GBSA binding free energies (ΔG_{bind}) for the top 50 machine learning–prioritized compounds after PAINS filtering, obtained from 50 ns molecular dynamics simulations. Error bars represent standard deviations calculated over 250 uniformly sampled trajectory snapshots. Energies were computed using the GBneck2 implicit solvent model in Amber without entropic correction.

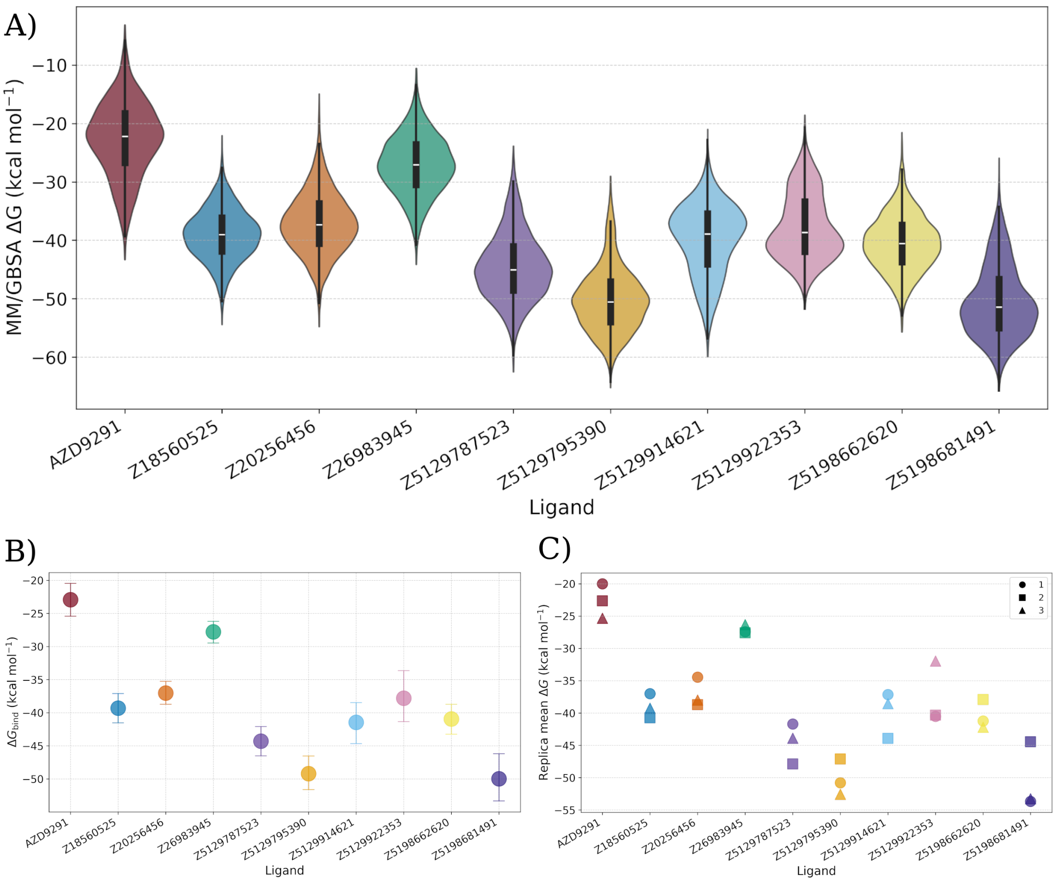

Figure S8. Statistical characterization of MM/GBSA binding free energy estimates obtained from replicate molecular dynamics simulations. (A) Violin plot showing the distribution of frame-wise MM/GBSA binding free energies (ΔG_{bind}) for each ligand across all analyzed trajectories, illustrating intra-trajectory energetic fluctuations. (B) Bootstrap-resampled estimates of the mean binding free energy, where error bars denote the 95% confidence intervals derived from statistically independent samples (N_{eff}), providing robustness assessment of ligand ranking. (C) Replica reproducibility analysis showing mean binding free energies obtained from independent simulation replicas, enabling evaluation of inter-replica convergence and sampling consistency.

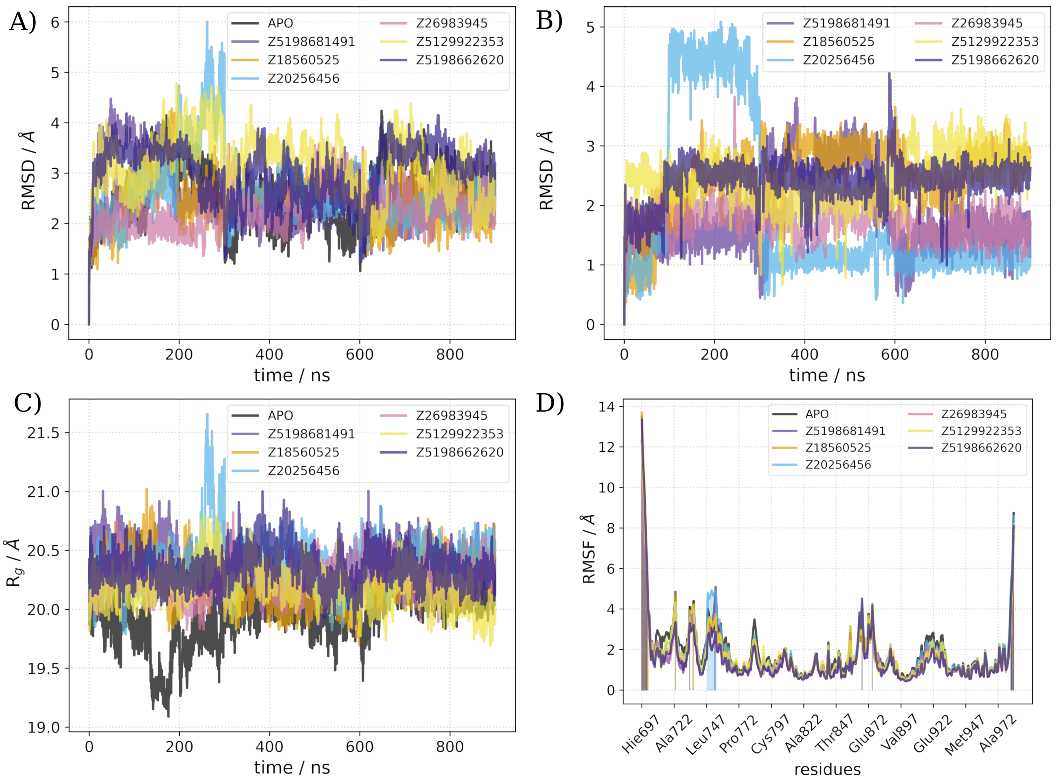

Figure S9. Structural stability analysis derived from 300 ns molecular dynamics simulations. Three independent MD replicates were concatenated into a single trajectory prior to analysis. (A) Root-mean-square deviation (RMSD) of the protein backbone atoms, (B) ligand RMSD relative to the initial bound conformation, (C) radius of gyration (R_g) of the protein, and (D) per-residue root-mean-square fluctuation (RMSF) calculated for heavy atoms. Amino acid residues exhibiting RMSF values exceeding 4 Å are highlighted to indicate regions of increased flexibility.

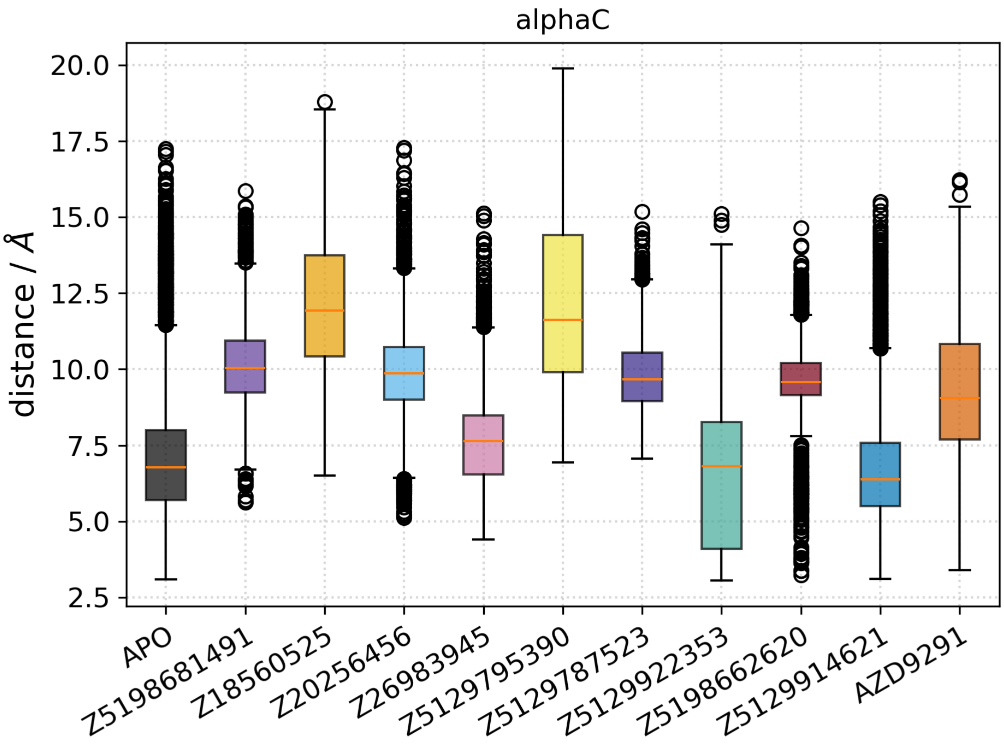

Figure S10. Boxplots of distances between the CD atom of Glu762 and the NZ atom of Lys745 across different ligands and the apo EGFR form. Each box represents the distribution of distances observed over the analyzed trajectory frames, with whiskers indicating the 1.5× interquartile range.

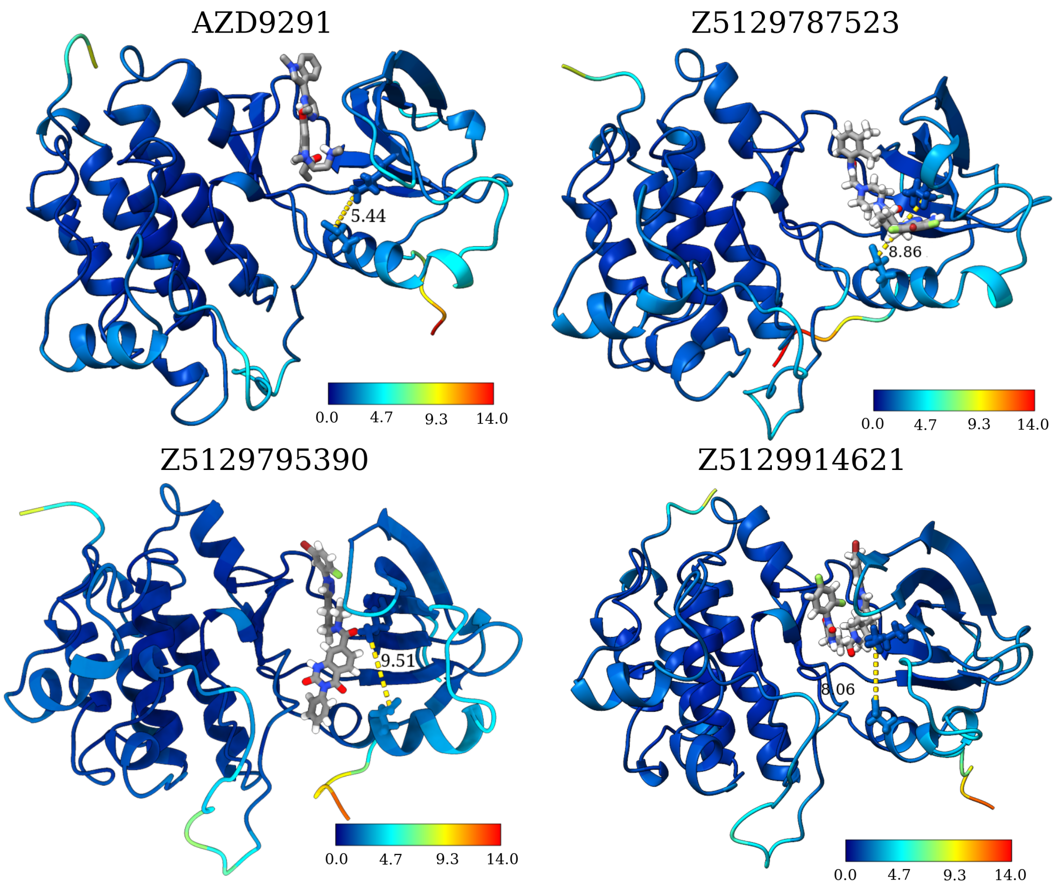

Figure S11. Terminal conformations of the four ligand–receptor complexes extracted from the molecular dynamics trajectories. The protein backbone is rendered in cartoon representation and coloured according to per-residue root-mean-square fluctuation (RMSF) values. The distance between the CD atom of Glu762 and the NZ atom of Lys745 is annotated for each complex (Å).

Table S1. Hyperparameter search ranges and corresponding optimal values identified through Optuna-based Bayesian optimization for SVM, RF, and XGBoost models. Reported optimal configurations correspond to the highest mean cross-validation score obtained under the composite objective penalizing overfitting.

	**SVM**	
**Hyperparameter**	**Search space**	**Optimal**
C	{"float", 10^{-2}, 10^{3}, "log"}	1.0236
epsilon	{"float", 10^{-3}, 1.0, "log"}	0.2904
gamma	{"float", 10^{-4}, 10^{-1}, "log"}	0.0562
kernel	{"cat", ["rbf"]}	rbf
	**RF**	
**Hyperparameter**	**Search space**	**Optimal**
n_estimators	{"int", 300, 800}	300
max_depth	{"int", 6, 20}	8
min_samples_split	{"int", 2, 20}	14
min_samples_leaf	{"int", 1, 10}	8
max_features	{"float", 0.3, 1.0}	0.4324
bootstrap	{"cat", [True, False]}	True
	**XGBoost**	
**Hyperparameter**	**Search space**	**Optimal**
n_estimators	{"int", 200, 300}	292
max_depth	{"int", 4, 6}	4
learning_rate	{"float", 0.03, 0.08, "log"}	0.0374
gamma	{"float", 0.08, 0.4}	0.3738
reg_alpha	{"float", 1.0, 10.0}	7.6377
reg_lambda	{"float", 1.0, 5.0}	1.0544
subsample	{"float", 0.7, 0.9}	0.7148
colsample_bytree	{"float", 0.7, 0.9}	0.7550
min_child_weight	{"float", 6.0, 15.0}	7.8975
max_delta_step	{"int", 3, 6}	6

Table S2. Summary of MM/GBSA binding free energy analysis for ligand–EGFR mutant complexes obtained from concatenated molecular dynamics trajectories. Reported quantities include mean binding free energy (ΔG_{bind}), inter-replica standard deviation ($\mathrm{SD_{br}}$), frame-wise fluctuation, statistical inefficiency (τ), effective sample size (N_{eff}), and bootstrap statistics.

Ligand	Z5198681491	Z5129795390	Z5129787523	Z5129914621	Z5198662620	Z18560525	Z5129922353	Z20256456	Z26983945	AZD9291
ΔG_{bind}	-50.5	-50.2	-44.5	-39.9	-40.4	-39.0	-37.6	-37.0	-27.1	-22.7
$\mathrm{SD_{br}}$	5.2	2.8	3.1	3.6	2.2	1.9	4.9	2.3	0.7	2.7
Frame fluct.	4.4	4.9	5.2	5.3	4.4	4.1	4.1	4.8	5.0	6.0
τ	461.5	236.8	193.5	230.0	175.0	176.6	428.5	229.8	128.4	209.6
N_{eff}	10	19	24	20	26	26	11	20	35	22
BootMean	-50.0	-49.2	-44.3	-41.4	-41.0	-39.3	-37.8	-37.0	-27.8	-22.9
BootSTD	1.8	1.3	1.1	1.6	1.2	1.1	2.0	0.9	0.8	1.3
CI_{low}	-53.3	-51.6	-46.5	-44.7	-43.2	-41.5	-41.4	-38.7	-29.5	-25.4
CI_{high}	-46.2	-46.6	-42.1	-38.5	-38.7	-37.1	-33.7	-35.3	-26.2	-20.5
